# *Ascophyllum nodosum* extract mitigates salinity stress in *Arabidopsis thaliana* by modulating the expression of miRNA involved in stress tolerance and nutrient acquisition

**DOI:** 10.1371/journal.pone.0206221

**Published:** 2018-10-29

**Authors:** Pushp Sheel Shukla, Tudor Borza, Alan T. Critchley, David Hiltz, Jeff Norrie, Balakrishnan Prithiviraj

**Affiliations:** 1 Marine Bio-products Research Laboratory, Dalhousie University, Department of Plant, Food and Environmental Sciences, Truro, Nova Scotia, Canada; 2 Research and Development, Acadian Seaplants Limited, Dartmouth, Nova Scotia, Canada; National Taiwan University, TAIWAN

## Abstract

*Ascophyllum nodosum* extract (ANE) contains bioactive compounds that improve the growth of *Arabidopsis* in experimentally-induced saline conditions; however, the molecular mechanisms through which ANE elicits tolerance to salinity remain largely unexplored. Micro RNAs (miRNAs) are key regulators of gene expression, playing crucial roles in plant growth, development, and stress tolerance. Next generation sequencing of miRNAs from leaves of control *Arabidopsis* and from plants subjected to three treatments (ANE, NaCl and ANE+NaCl) was used to identify ANE-responsive miRNA in the absence and presence of saline conditions. Differential gene expression analysis revealed that ANE had a strong effect on miRNAs expression in both conditions. In the presence of salinity, ANE tended to reduce the up-regulation or the down-regulation trend induced caused by NaCl in miRNAs such as ath-miR396a-5p, ath-miR399, ath-miR2111b and ath-miR827. To further uncover the effects of ANE, the expression of several target genes of a number of ANE-responsive miRNAs was analyzed by qPCR. NaCl, but not ANE, down-regulated miR396a-5p, which negatively regulated the expression of *AtGRF7* leading to a higher expression of *AtDREB2a* and *AtRD29* in the presence of ANE+NaCl, as compared to ANE alone. ANE+NaCl initially reduced and then enhanced the expression of ath-miR169g-5p, while the expression of the target genes *AtNFYA1* and *ATNFYA2*, known to be involved in the salinity tolerance mechanism, was increased as compared to ANE or to NaCl treatments. ANE and ANE+NaCl modified the expression of ath-miR399, ath-miR827, ath-miR2111b, and their target genes *AtUBC24*, *AtWAK2*, *AtSYG1* and *At3g27150*, suggesting a role of ANE in phosphate homeostasis. *In vivo* and *in vitro* experiments confirmed the improved growth of *Arabidopsis* in presence of ANE, in saline conditions and in phosphate-deprived medium, further substantiating the influence of ANE on a variety of essential physiological processes in *Arabidopsis* including salinity tolerance and phosphate uptake.

## Introduction

Environmental stresses such as increased soil and water salinity, drought and heat have detrimental effects on agricultural productivity [1]. Over one-third of the total global, arable land, or nearly 950 million hectares, is affected by high saline conditions [2,3]. Salinity and other abiotic stresses negatively influence the mineral-nutrient relations in plants [4]. Plants are resilient organisms and are equipped with a wide array of adaptations which help them to tolerate perturbations caused by such stresses. The response of plants to salinity stress is known to be a complex and dynamic process that is controlled by multiple genetic loci [1]. The most noticeable adaptations include increased expression of stress-responsive genes and of various transporters, the production of protective metabolites such as osmolytes and polyamines and of antioxidative enzymes [1,2,5]. The transcriptional machinery associated with stress responses regulates aspects of plant growth, metabolism and development through the maneuvering of an intricate network of transcription factors [1]. In addition, post-transcriptional and post-translational modifications such as ubiquitination and sumoylation play an important role in the regulation of gene expression during stressful conditions and, when taken together, these strategies help plants to adapt and survive certain levels of salinity stress [6].

In recent years, transcriptome analysis using high-throughput sequencing revealed that approximately 90% of eukaryotic genomes are transcribed into RNAs, although only a small number correspond to protein-coding RNA [7]. Non-coding RNAs (ncRNAs) comprise a diverse group of transcripts, including house-keeping ncRNAs (ribosomal RNAs, transfer RNAs, small nuclear RNAs and small nucleolar RNAs) and many regulatory ncRNAs. Non-coding RNAs are classified into small ncRNA (20–30 bp) and large ncRNA (more than 200 bp; [8]). Transcriptional gene silencing is mediated by small RNA, such as micro RNAs (miRNAs) and small interfering RNAs (siRNAs), which play an important role in the post-transcriptional regulation of eukaryotic gene expression that is required by many fundamental biological processes [9]. These small RNAs are known to silence genes post-transcriptionally by guiding target mRNA for degradation or by repressing translation [10]. Several miRNAs, responsive to various abiotic stressors, have been identified from plant species of economic importance, e.g. rice, barley, wheat, sugar cane, legumes, tomato, potato and others [11–21]. Various miRNAs have been reported to be induced by drought and salinity stresses in different plant species, e.g. miR156, miR159, miR165, miR167, miR168, miR169, miR319, miR393, miR395, miR396, miR398, miR399 and miR402 [22,23]. In *Arabidopsis*, several miRNAs have been also described to regulate phytohormone signaling during exposure to abiotic stress [24]. Thus, analyzing the pattern of miRNAs expression represents a novel tool to further aid our understanding of the complexity of the varied stress tolerance mechanisms in plants.

Due to the complex metabolic pathways involved in salinity tolerance, there has been limited success in generating salt-tolerant crop varieties through genetic engineering. Additionally, transgenic approaches face limitations such as meeting regulatory demands and consumer approval. A different approach to improve stress tolerance in plants is the use of plant biostimulants [25–27]. Macroalgae, such as *Ascophyllum nodosum* (rockweed), have been explored as a commercial source of varied bioeffectors and of biostimulants for plant growth [28,29]. According to Van Oosten et al. (2017) [28], nearly 47 companies are currently involved in the preparation of commercial extracts from *A*. *nodosum* for agricultural applications. Brown seaweeds in general (either whole as meal or as powder/liquid extracts), are known to possess plant growth-promoting activity and are widely used for agricultural and horticultural crop production [29]. *Ascophyllum nodosum* extract (ANE) has been demonstrated to promote root and shoot growth of *Arabidopsis*, which can be considered as a model organism representative of many crop plants, by regulating the phytohormone metabolism of treated plants [30,31]. ANE and its lipophilic fraction enhanced freezing tolerance in *Arabidopsis* by modulating the expression of freezing-stress responsive genes involved in the accumulation of osmoprotectants [32,33]. Treatment with ANE were found to increase the content of carbon, nitrogen and leaf chlorophyll [34], to augment water retention capacity and to ameliorate certain abiotic stresses [35]. Bioactive compounds present in a commercial extract of *A*. *nodosum* were shown to improve plant growth under saline stress (150 mM NaCl) [36]. *A*. *nodosum* extracts applied to grasses also improved tolerance to drought stress [37]. In the present study, using next generation sequencing, several miRNAs involved in *A*. *nodosum* extract (ANE)-mediated salinity tolerance in *Arabidopsis* were identified. In addition, the expression pattern of several genes which are targeted by the miRNA and whose expression was found to be modulated by ANE, was analyzed by quantitative real-time PCR. Data from the two approaches revealed that ANE influenced the regulation of expression of many stress-responsive genes.

## Materials and methods

### Treatment of *Arabidopsis* with ANE under experimentally induced saline condition

*Arabidopsis thaliana* Col-0 seeds were purchased from Lehle Seeds (Round Rock, TX, USA). A specific *Ascophyllum nodosum* extract (ANE), commercially available as Acadian Marine Plant Extract Powder, produced in August 2015 by Acadian Seaplants Ltd., Dartmouth, Nova Scotia, Canada, was used in this study. The source of the brown alga *Ascophyllum nodosum* was Annapolis Basin, Nova Scotia, Canada, and processing was carried out in Cornwallis, Nova Scotia, Canada. Entire plant was ground, and soluble compounds extracted using an alkaline (KOH) extraction method. After extraction and spray-drying the product was stored at 4°C until use. The chemical composition of the product was described elsewhere [33]. Other information regarding this product can be found at: http://www.acadianseaplants.com/plant-growth-development-products/direct-plant-applications/labels.

In order to evaluate the in *vivo* effects of ANE on *Arabidopsis*, in the absence and in the presence of salinity stress, seeds were germinated on peat pellets (Jiffy-7W, Jiffy Products Ltd, NB, Canada) in a growth chamber with controlled conditions: 22/18°C day/night temperatures and 16/8 h photoperiod with an irradiance of 100 μmol photons m^-2^s^-1^. The experiment was designed to assess the effects of salinity stress (150 mM NaCl) in the absence and in the presence of ANE (0.1% w/v), as described by Jithesh et al. (2012) [27], with minor modifications. In addition to the control (water-treated, C), the other three conditions were ANE-treated (T_1_), ANE+NaCl-treated (T_2_) and NaCl-treated (T_3_) plants. ANE (0.1% w/v) and NaCl (150 mM) were applied to three-week-old plants by irrigation, at the rate of 20 mL per plant every week for three weeks (S1 Fig). Plants treated with water or with NaCl, grown under identical experimental conditions, served as control (C) or were NaCl-treated (T_3_), respectively. After three weeks of treatments, plants were dried at 72°C for 72 h. Na^+^, K^+^, and P content was measured from the dried acid-digested plant samples with an inductively coupled plasma optical emission spectrometer (ICP-OES), at the Analytical and Dairy Lab, Harlow Institute, Nova Scotia, Canada. RNA was extracted from whole 3 three-week-old plants that were harvested 6 h and 12 h post-treatment and frozen in liquid nitrogen. All treatments were performed in triplicate (three biological replicates) and each replicate contained 3 plants.

To study the *in vitro* root system architecture, seeds were surface sterilized with 2.5% Chlorox and stratified at 4 °C in the dark for 72 h. Seeds were germinated on ½ MS medium [38] supplemented with 1% sucrose and 0.8% Phytagel (Sigma, USA), at pH 5.8. Four-day-old seedlings were transferred to square Petri dishes with a grid containing ½ MS medium. The effect of 0.01% ANE [31] on the root system architecture of *Arabidopsis* was also evaluated on ½ MS phosphate-depleted medium (MSP19, Caisson Labs, USA), in presence of 100 mM NaCl, and of both stresses together (i.e., no phosphate and salinity stress). Plates were placed in the growth chamber with controlled conditions: 22/18 °C day/night temperatures and 16/8 h photoperiod with an irradiance of 100 μmol photons m^-2^ s^-1^. The increase in length of the primary root and the number and length of lateral roots were recorded for 7 days after the imposition of salinity stress, phosphorus starvation, and both of these stressors in combination. The data for the *in vitro* analyses was generated using 6 square plates with 4 plants/ plate for each experimental condition and the experiment was repeated 3 times.

### Isolation of RNA and small RNA library preparation

Total RNA was extracted using miRNAeasy kit (Qiagen, Germany) using manufacturer’s protocol. The quantity and purity of the total RNA was determined using a NanoDrop2000 spectrophotometer (Thermo Scientific, USA) and an Agilent 2100 bioanalyser (USA) with the RNA Integrity Number (RIN) > 8.0. The extracted total RNA was stored at -80°C, and was used for high-throughput next generation sequencing and real-time quantitative gene expression analysis.

Small RNA libraries were prepared using 1 μg of RNA (mixed from three plants) and the TruSeq Small RNA Library Preparation Kit (Illumina, USA). For miRNAs, the sRNA libraries were prepared from mature miRNA which resulted from the cleavage by DICER enzymes, and the reverse transcription reaction was performed to prepare single stranded cDNA. The cDNA was size fractionated to obtain miRNA and sequencing was performed using the Illumina HiSeq 2500 machine by Genome Quebec, Montreal, Canada. A total number of 24 miRNA libraries were sequenced. The sequenced libraries were represented by biological triplicates of control and of the three treatments, ANE (T_1_) ANE+NaCl (T_2_) and NaCl (T_3_) at two time points (6 h and 12 h).

### Data processing, annotation and identification of miRNA

After Illumina sequencing, the raw sequencing reads from the control, ANE, ANE+NaCl and NaCl treated samples were trimmed by removing the adaptor sequence (TGGAATTCTCGGGTGCCAAGG), sequences in length greater than 18–25 nucleotides, and tRNA contamination. The filtered reads from each of the samples were mapped to the *Arabidopsis* genome (The Arabidopsis Information Resource release version 10; http://www.arabidopsis.org) using the mirAligner program. Subsequently, the reads were mapped to the mature miRNA sequences obtained from miRbase (release 21.0). The relative frequencies of each miRNA family in the different treated libraries were indicated in terms of count per million.

### Differential expression analysis of ANE-induced miRNAs involved in salinity tolerance of *A*. *thaliana*

In order to determine the expression of each miRNA in different treatments, the normalization factor for each library was calculated using DESeq [39], implemented in the R statistical programming environment. All miRNAs were normalized to a transcript expression level per million counts. Differentially expressed miRNAs were determined through fold change. The threshold set up for up and down regulated genes was a fold change of ≥ 1.2 in at least one of the treatments and one of the time points and a *p* value ≤ 0.05. This threshold cut-off is similar to that considered significant in a miRNA expression study of cotton [40] and is slightly less stringent compared to other miRNA differential expression studies [41–43].

### Prediction of genes targeted by ANE-induced miRNAs involved in the salinity of *A*. *thaliana*

psRNATarget server (http://plantgrn.noble.org/psRNATarget/) and AtMiRnet (http://atmirnet.itps.ncku.edu.tw/) were used to predict miRNA targets, and to further determine if the expression of these genes was also modulated by the application of ANE, in the presence and absence of salinity stress. The parameters for the target prediction were the default settings. To increase the specificity of predictions only those target predictions suggested by both methods employed were considered for further expression analysis. The targets of miRNAs were predicted based on scoring for reverse complementarity and target-site accessibility by calculating the unpaired energy required to open secondary structures around small RNA target sites on mRNA [44].

### Validation of expression of target mRNA through real-time quantitative PCR

Real-time quantitative PCR was used for determining the expression of target genes for which gene specific primers have been designed using the sequences available in GenBank (S1 Table). For the validation of target genes, samples were harvested at 6 and 12 h post-treatment from control and the three treatments. These samples originated from the same experiment that was used for NGS of miRNAs. Total RNA was extracted using the RNAeasy kit (Qiagen, Germany), following manufacturer’s protocol. The quantity and purity of the total RNA were analyzed using a NanoDrop 2000 spectrophotometer (Thermo Scientific, USA). An amount of 2.5 μg RNA from each sample was treated with DNase I (Promega, USA) and then the synthesis of cDNA was performed using the Revert Aid cDNA reverse transcription kit (Thermo Scientific, USA). Real-time quantitative PCR (qPCR) was performed using the StepOne Plus Real-Time PCR system (Applied Biosystems). Actin was used as a reference gene. The specificity of PCR amplification was checked at the end of the PCR cycles by melt-curve analysis. Each biological sample had three technical replicates and the relative-fold expression was determined using the Livak (2^-ΔΔ Ct^) method [45].

### Statistical analysis

Data were analyzed using ANOVA, with a p-value of ≤ 0.05 using the “Proc. mixed procedure”, of the SAS Institute, Inc. Software version 9.3 (SAS Institute, Inc., Cary, NC, USA). When significant effects of treatments were found, multiple means comparison was carried out using Tukey’s analysis with a 95% confidence interval.

## Results

### ANE improved growth and root system architecture of *Arabidopsis* in the presence of a saline stress

**Fig 1:**
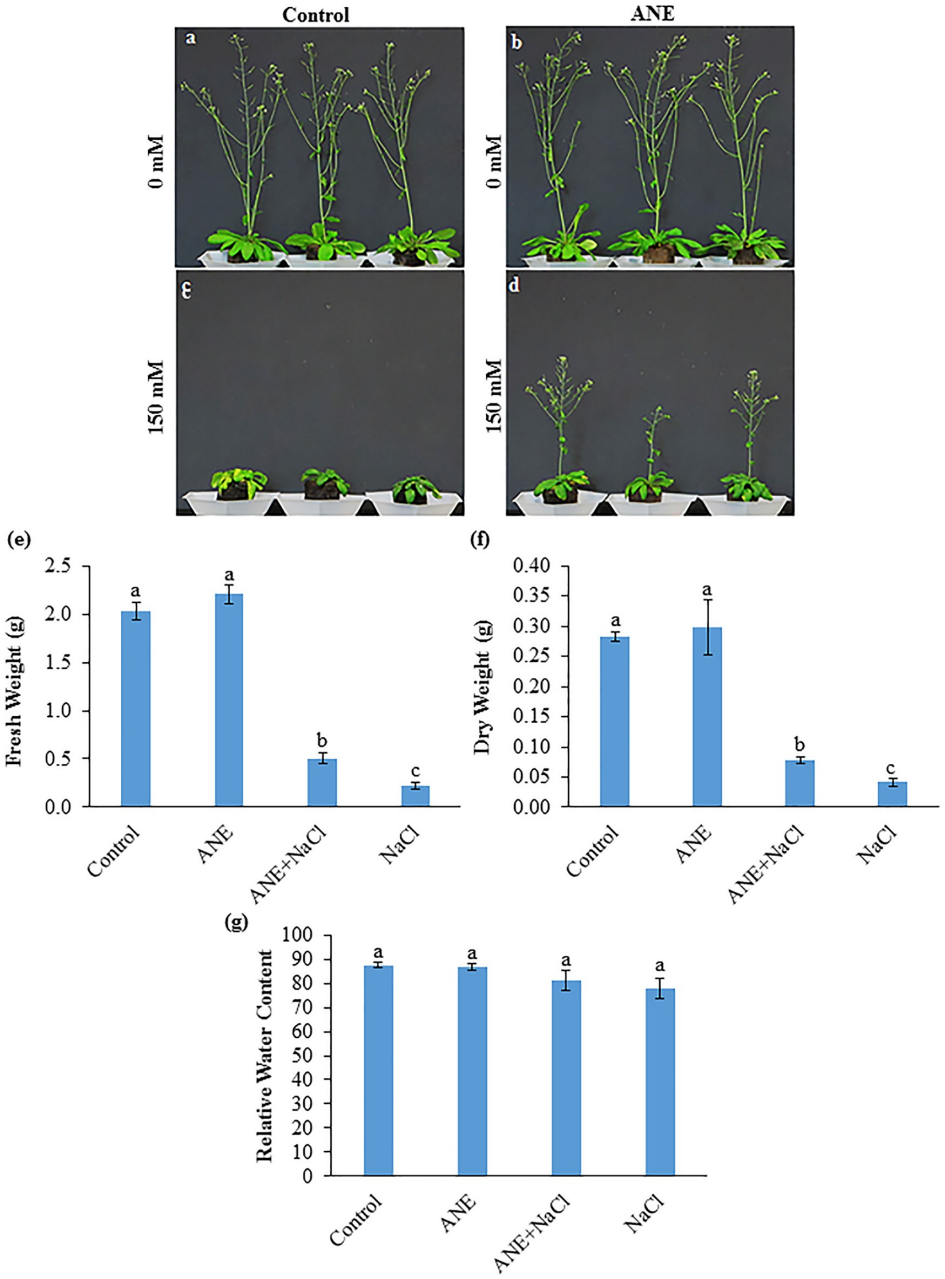
ANE improves the growth of *Arabidopsis thaliana* in the presence of salt stress. Seven-week-old *Arabidopsis* plants grown on Jiffy pellets, 28 days after treatments Control **(a)**, 0.1% ANE (**b)**, 150 mM NaCl **(c)**, and 0.1% ANE and 150 mM NaCl (**d)**. The effect of the ANE on the fresh **(e)** and dry weight **(f)** and relative water content **(g)** of *Arabidopsis* growing in the presence and absence of 0 and 150 mM NaCl.

The application of ANE improved the growth of *Arabidopsis* on peat pellets as well as in Petri dishes in the presence of salinity (Fig 1a–1d; Parts a-d in S2 Fig). *Arabidopsis* grown on peat pellets showed higher fresh and dry weight, but not relative water content in the presence of ANE+150 mM NaCl, as compared to the plants treated with NaCl alone (Fig 1g and 1f). ANE supplemented *Arabidopsis* plants showed significantly lower Na^+^ and higher P content in the presence of NaCl as compared to the plants grown in presence of NaCl alone (S2 Table). K^+^/Na^+^ ratio was also higher in ANE+150 mM NaCl, treatment as compared to the plants treated with NaCl alone. In *in vitro* experiments ANE-treated *Arabidopsis* seedlings showed a significant increase in fresh and dry weight, in the presence 100 mM NaCl (Parts e-f in S2 Fig), as compared to the control plants treated with 100 mM NaCl. The application of ANE also resulted in enhanced relative water content in those seedlings stressed with 100 mM NaCl (Part g in S2 Fig). Furthermore, ANE applications improved root growth and development in the presence of salinity stress (S3 Fig). Seven days post-treatment, the seedlings treated with 100 mM NaCl, in the presence of ANE had, in average, longer primary roots, with an enhanced number and length of lateral roots, as compared to control seedlings stressed with NaCl alone (Parts a-c in S3 Fig).

### High-throughput sequencing of control, ANE, ANE + NaCl and NaCl treated small RNAs

The depth of sequencing using the Illumina platform varied from 8471991 to 17517592 reads (average of the raw reads from three replicates) (Part a in S4 Fig). Raw reads were trimmed with cut-adapt and after removing the low-quality reads, the number of sequences reads and unique sequences from the raw data were calculated and mapped on the *Arabidopsis thaliana* MIRBase (release 21.0) using mirAligner. A number of 2146618, 2477588, 2567517 and 3210249 reads were mapped to the genome, with a perfect match, to the libraries prepared at 6 h from control, ANE, ANE + NaCl and NaCl, respectively; at 12 h the corresponding number of reads were 1862236, 2275517, 3394958 and 4589656 (Part b in S4 Fig).

The mapped sequences from different libraries, with lengths ranging from 15–25 nucleotides, were used to examine the correlation between the length of miRNAs and the proportion of total sequence reads. At 6 h, the most abundant size of miRNA in the control libraries was 20 nucleotides (46.63%), followed by 19 nucleotides (17.52%) and 21 nucleotides (11.06%). The small RNA distribution pattern was observed to be the same in ANE- and ANE+NaCl-treated libraries after 6 h, while in the NaCl library, small RNA with 20 nucleotides (55.99%) was the most abundant, followed by 19 nucleotides (17.93%) and 21 nucleotides (13.53%). At 12 h time point, the control, ANE, ANE+NaCl and NaCl samples showed the similar distribution patterns, with the most abundant size of miRNA being 20 nucleotides, followed by 19 and 21 nucleotides. These results suggested that treatment with ANE did not affect the differential distribution patterns of the sizes of small RNAs (S5 Fig).

To determine the number of known micro RNAs in different libraries at 6 and 12 h, all the unique sequences with lengths between 15–25 nucleotides were aligned to all annotated miRNA precursors in *Arabidopsis* (miRbase release 21.0). In order to compare the miRNA abundance at 6 and 12 h, the profusion of each micro RNA in a library was normalized using DESeq software. miRNAs with less than 1 CPM in each library were excluded in the DGE analyses. In all libraries, including 6 and 12 h, miR166a-3p was the most abundant, followed by the ath-miR158, ath-miR165, ath-miR398, ath-miR159 and ath-miR319 (see S3 and S4 Tables for details).

### Differentially expressed micro-RNAs, in response to ANE and ANE-induced salinity tolerance in *A*. *thaliana*

**Fig 2:**
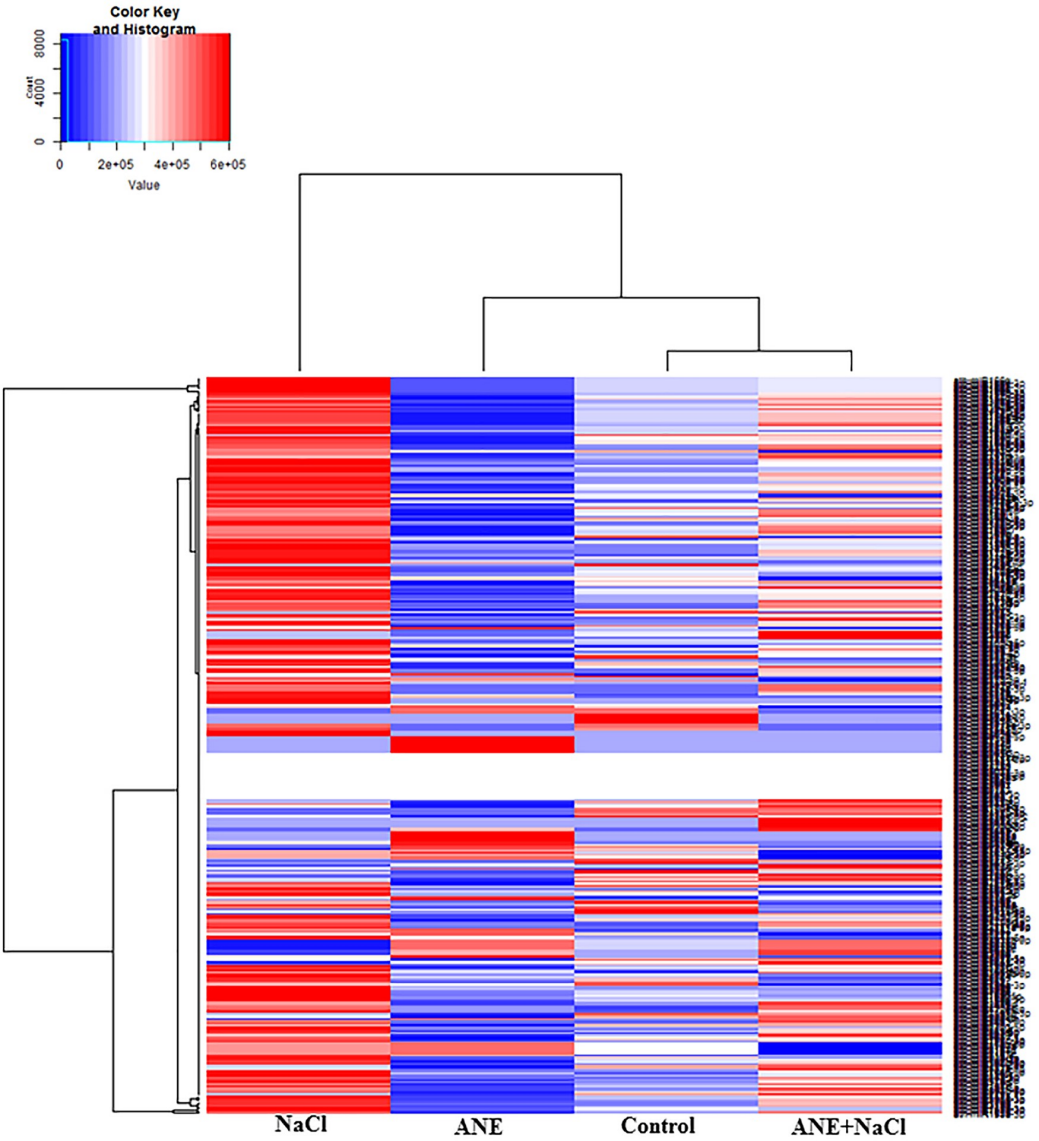
Cluster analysis of gene expression in *Arabidopsis* in the control and in presence of ANE, ANE+NaCl and NaCl at 6 h of the treatment. The heat map was generated based on hierarchical analysis using the average of CPM from three replicates of *Arabidopsis* from control, ANE, ANE+NaCl and NaCl libraries. The colors ranging from blue to red, represent the average values of CPM.

**Fig 3:**
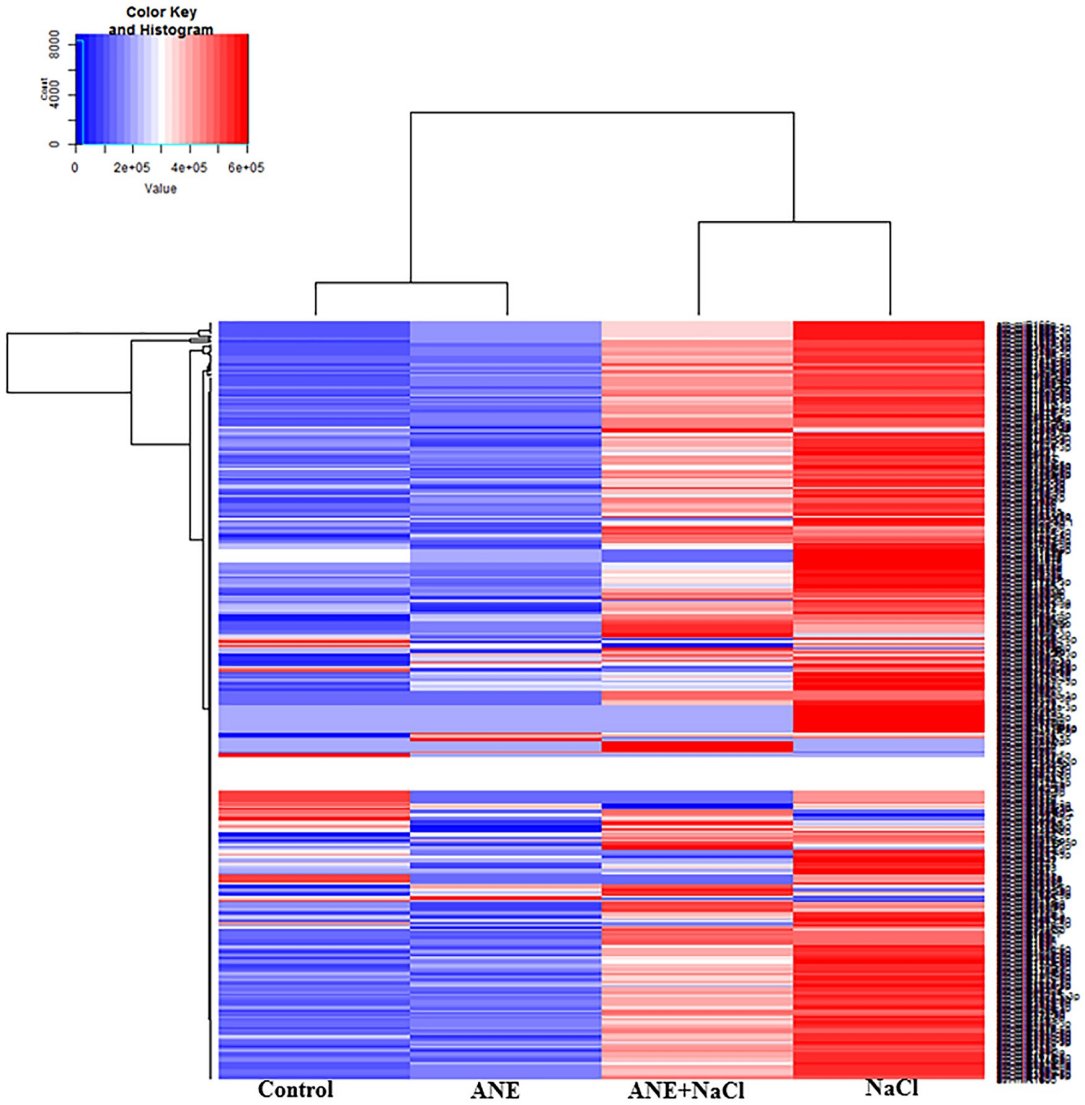
Cluster analysis of gene expression in *Arabidopsis* in the control and in presence of ANE, ANE+NaCl and NaCl at 12 h of the treatment. The heat map was generated based on hierarchical analysis using the average of CPM from three replicates of *Arabidopsis* from control, ANE, ANE+NaCl and NaCl libraries. The colors ranging from blue to red, represent the average values of CPM.

**Fig 4:**
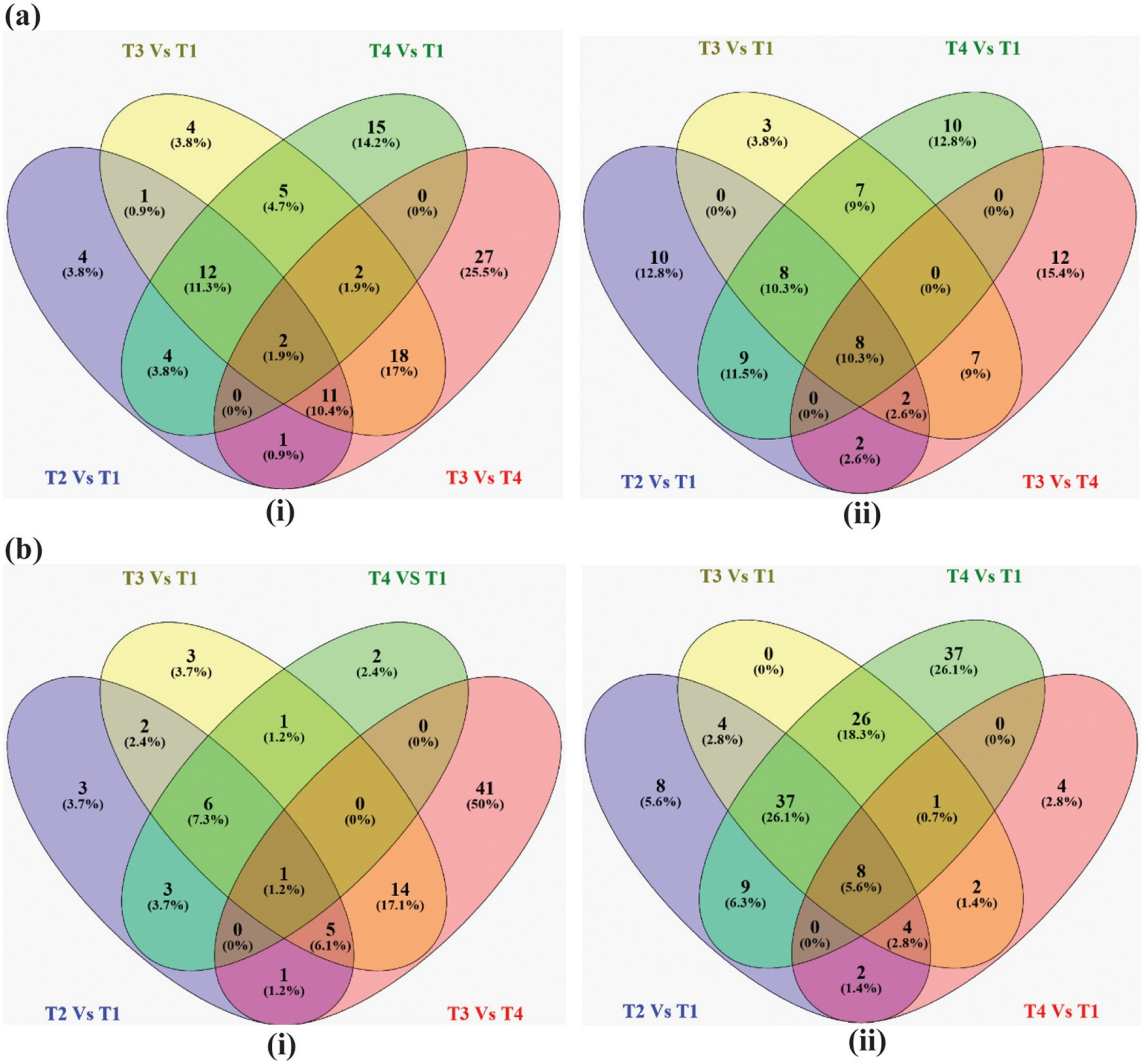
Venn diagrams of differentially expressed miRNAs. (a) Venn diagrams showing the up-regulation (i) and down-regulation (ii) of miRNAs in the libraries of *Arabidopsis* from control (C) and the 3 treatments: ANE (T_1_), ANE+NaCl (T_2_), and NaCl (T_3_) at 6 h. (b) Venn diagrams showing the up-regulation (i) and down-regulation (ii) of miRNAs in the libraries of *Arabidopsis* from control (C) and the 3 treatments: ANE (T_1_), ANE+NaCl (T_2_), and NaCl (T_3_) at 12h.

ANE-responsive miRNAs were systematically identified by analysis of differential expression patterns between the different treatments at 6 and 12 h time points. MA plots represented the differential expression pattern for control and the treatments (S6 and S7 Figs). In total, 106 miRNAs were found to be differentially expressed (>1.2-fold change) in at least one of the treatments and one of the time points. Many miRNAs were found to be differentially expressed after ANE treatments or by ANE in the presence of salinity stress. These changes in the abundance of miRNAs revealed specific patterns of expression in control and in the ANE, ANE+NaCl and NaCl-treated libraries, at different time points. At 6 h, the expression pattern of microRNA in control, ANE and ANE+NaCl displayed more similarities therefore they clustered together, clearly separated from NaCl (Fig 2). The hierarchical clustering of miRNA reads at 12 h revealed that the miRNA expression patterns in ANE+NaCl and NaCl-was more similar and, therefore, they were grouped together, whilst the control and ANE treatments formed a different cluster (Fig 3). Six hours after ANE application, from the total of 106 miRNAs, 16 miRNAs were up-regulated while 23 miRNAs were down-regulated. At 12 h after the application of ANE, 12 miRNAs were up-regulated, and 34 miRNAs were down-regulated. In presence of salinity stress ANE treatment determined more pronounced changes in the miRNAs expression, with 22 miRNAs up-regulated and 25 miRNAs down-regulated after 6 h, and 17 miRNAs up-regulated and 46 miRNAs down-regulated after 12 h. As expected, salinity stress (NaCl alone) strongly influenced the expression of many miRNAs. At 6 h, 18 miRNAs were up-regulated and 28 miRNAs were down-regulated while 12 h after the application of salinity stress 8 miRNAs were up-regulated and 55 miRNAs were down-regulated. Among them, after 6 h of NaCl treatment, 14.2 and 12.8% of the miRNAs were found to be up-regulated and down-regulated, respectively, as compared to control, while in the ANE+NaCl treatment, 25.5 and 15.4% of miRNA were up-regulated and down-regulated, respectively, as compared to the NaCl (Fig 4a). After the 12 h treatment period and amongst those differentially expressed miRNAs, 50% were up-regulated by ANE applications in the presence of a saline stress as compared to the plants treated with NaCl whilst only 2.4% of miRNAs were up-regulated in those plants treated with NaCl, as compared to control (Fig 4b). Conversely, only 2.8% of miRNA were down-regulated in the plants treated with ANE+NaCl, as compared to the plants treated with NaCl, while 26.1% of miRNAs were down-regulated (Fig 4b).

**Fig 5:**
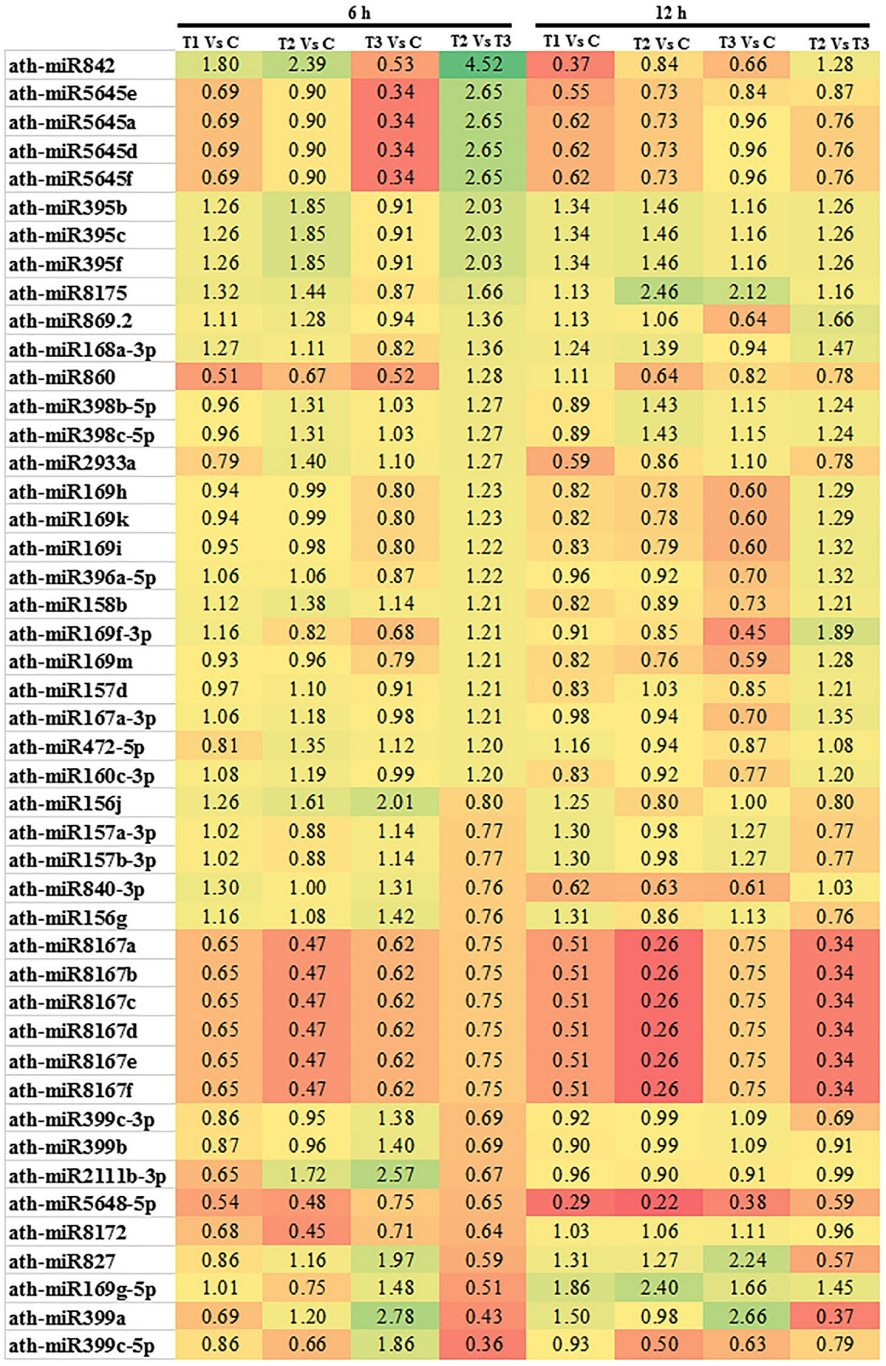
ANE modulated the expression of miRNA in *Arabidopsis* treated with NaCl. Differentially expressed miRNA in *Arabidopsis* from control (C) and the 3 treatments, ANE (T_1_), ANE+NaCl (T_2_), and NaCl (T_3_) at 6 and 12 h. Differential expression of miRNA was represented in terms of fold change. The threshold set up for up and down regulated genes was a fold change of ≥ 1.2 and a p value ≤ 0.05. The colors ranging from red to green, represent the average values of fold change (red, low expression and green, high expression).

Among the differentially expressed miRNA, 6 h after the treatment, ath-mir842 was up-regulated (1.80-fold change) by ANE whilst ANE in the presence of NaCl induced even more (2.39-fold change). In contrast, for the same time point, salinity stress (NaCl) effected a reduction in the expression of this miRNA (Fig 5). The differential expression of ath-mir842 in the two treatments involving NaCl suggests that ANE-induced the expression of this miRNA in spite of an opposite trend determined by salinity. However, after 12 h this tendency was reverted and the abundance of ath-mir842 in the ANE and ANE+NaCl treatments was found to be much lower than in the control (Fig 5). After 6 h and 12 h, ANE-induced the expression of ath-miR395 (b, c and f) in both conditions while NaCl had almost no effect (Fig 5). The expression of ath-miR8175 was also increased by ANE and this effect was augmented by salinity conditions, from 1.44 to 2.46 times at 6–12 h, respectively. In the presence of salt stress ath-miR8175 expression increased significantly from 0.87 to 2.12 times, from 6 h to 12 h as compared to the control (Fig 5). No significant changes in transcript abundance of ath-miR869.2 were observed from 6 h to 12 h in the ANE-treated plants (Fig 5). Salinity stress, however, reduced the expression of this miRNA from 0.94 to 0.64 times from 6 h to 12 h as compared to control, leading to a difference of 1.36 to 1.66 times at 6 h to 12 h, respectively, between ANE+NaCl and NaCl alone (Fig 5).

The expression of ath-miR860 was reduced by ANE at 6 h (2 fold decrease in expression) but this effect was reverted after 12 h (the expression was 1.11 of the control). However, under saline stress ANE reduced the expression of ath-miR860 at both time points and a similar trend was observed in the NaCl treatment (Fig 5). ANE slightly induced the expression of ath-miR398b-5p and c-5p in the salt-stressed plants at both 6 and 12h, as compared to ANE and the NaCl treated plants (Fig 5). Similar expression patterns were observed in the case of ath-miR399a, ath-miR399b, ath-miR399c-3p and ath-miR827 with moderate effects from ANE and pronounced activation by salinity (NaCl alone) at one or both time points (Fig 5). An interesting expression pattern was observed for ath-miR169g-5p. At 6 h the expression of this miRNA was not influenced by ANE or slightly inhibited by ANE+NaCl. In contrast, after 12 h, ANE and ANE+NaCl induced ath-miR169g-5p, 1.8 times and 2.4 times, respectively. Notably, salinity stress moderately enhanced the expression of ath-miR169g-5p at both time points, suggesting again that ANE can differentially modulate miRNA expression in presence of salinity stress (Fig 5).

### Identification and validation of target genes

A crucial step in understanding the way ANE mitigates salinity stress in *Arabidopsis* is the identification of the target genes of miRNAs and analysis of their expression pattern. The targets of plant miRNA can be predicted *in silico*, on the basis of sequence similarity, since miRNAs usually show high sequence complementarity to their targets, as a mechanism of post-transcriptional regulation. To better understand the functionality of *A*. *nodosum* extract-induced miRNAs in this study, putative targets were predicted using two computational programs, psRNA target [44] and AtmiRNET [46]. The potential targets of the ANE-induced miRNAs were predicted with a 0.0–3.0 expectation-score cut-off threshold. The potential target genes appeared to be involved in a broad range of biological processes (S5 Table). Targets of most miRNAs induced by ANE or by ANE-mediated salinity tolerance in *Arabidopsis* include gene-encoding transcription factors such as ath-miR396 which targets growth-regulating transcription factor (GRF), ath-miR156g which targets squamosal promoter-binding (SBP)-like transcription factors, ath-miR169 which targets nuclear factor Y, CCAAT-binding factor and HAP2-like transcription factors, ath-miR2111b which targets MYB and NAC transcription factors and ath-miR863 which targets *AtMYB66* and nuclear-localized R3-type MYB transcription factors. These examples support the hypothesis that the miRNAs induced by ANE were involved in the regulation of a large gene expression network. In addition to the transcription factors, other genes also were found to be regulated by miRNAs induced by application of ANE, i.e. ath-miR398 targets superoxide dismutase 1 (*AtCSD1*) which is involved in the detoxification from superoxide radicals, ath-miR168a which targets *ARGONAUTE 1* that is involved in cross-talk between auxin and light signaling during adventitious root development, and ath-miR399a which targets ubiquitin-conjugating E2 enzyme (*AtUBC24*) and wall-associated kinase 2 (*AtWAK2*).

### Expression of target genes

**Fig 6:**
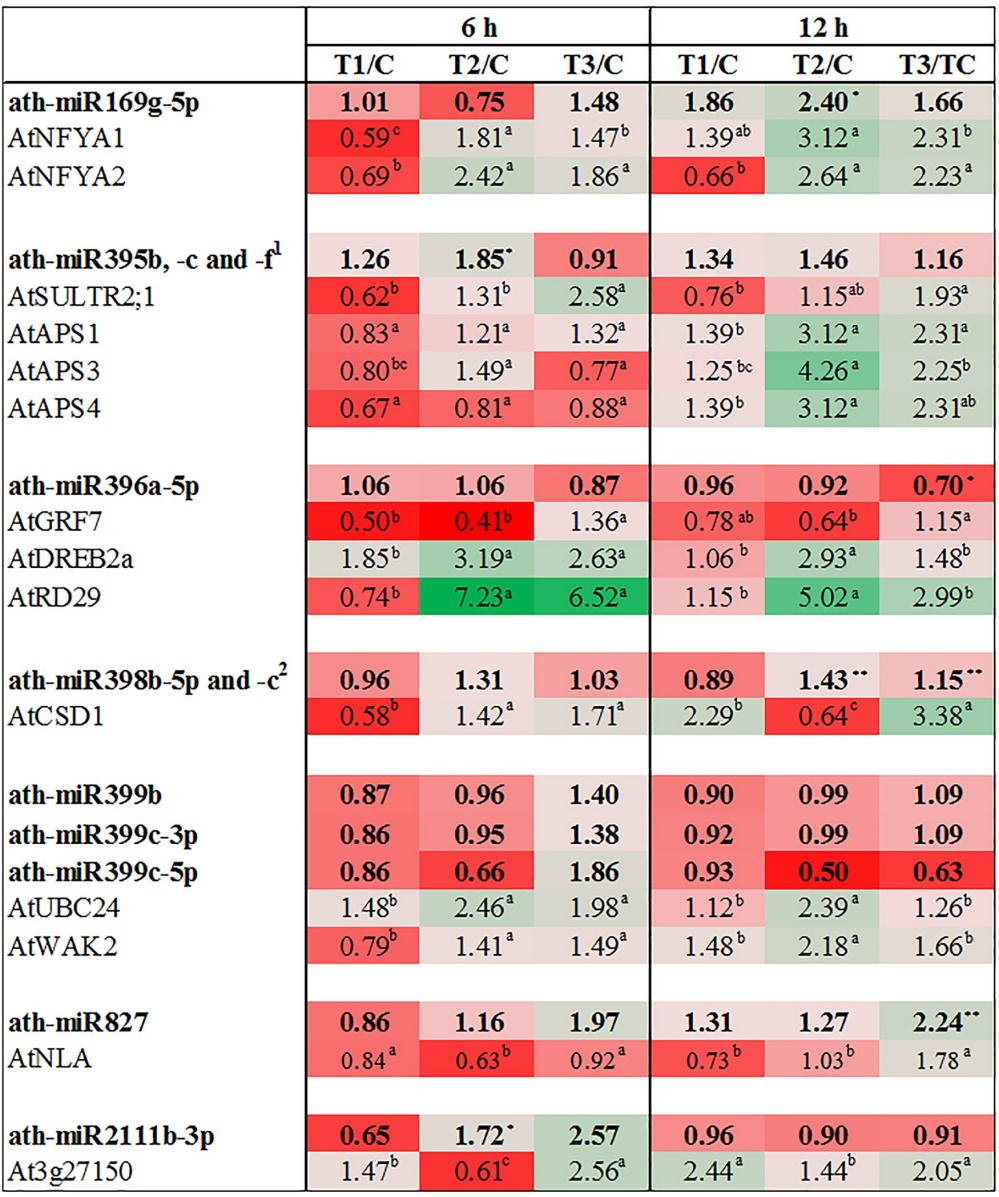
The effects of ANE in normal and salinity conditions and of NaCl on miRNA and on their target genes. Fold change of miRNAs (bold) and the relative expression of their target genes from control (C) and the three treatments: ANE (T_1_), ANE+NaCl (T_2_), and NaCl (T_3_) at 6 and 12 h. The colors ranging from red to green, represent the average values of fold change (red, low expression and green, high expression).

To investigate the function of miRNAs in regulating the expression of the predicted target genes after ANE treatment and in the ANE-mediated salinity tolerance in Arabidopsis, the expression of the target genes was studied by real-time quantitative PCR. Growth-regulating factors (GRF) belong to the transcription factor family that regulates growth and development of plants [15]. Real-time quantitative PCR expression analysis of *AtGRF7*, a target of ath-miR396a, showed a reduction of its expression level in the ANE-treated plants both in the absence or presence of NaCl, after 6 and 12 h, as compared to the plants treated with only NaCl (Fig 6). *AtGRF7* is known to negatively regulate the expression of *AtDREB2a* and *AtRD29* [47]. The expression of *AtDREB2a* and *AtRD29* was found to be higher in the ANE+NaCl-treated plants as compared to the NaCl-treated plants and these differences were statistically significant 12 h after the treatments (Fig 6).

In a previously published report, the transcription factor *NFYA* (Nuclear Factor Y, subunit A) has been identified as an miR169 target [48]. At 6 h the expression of miR169g-5p was not modified by the addition of ANE, was suppressed by the ANE+NaCl treatment, and was elevated by the addition of NaCl. At 12 h transcript abundance of this miRNA was increased in all three treatments as compared to control (Fig 6). The expression of the target genes, *AtNFYA1* and *AtNFYA2*, was high in saline conditions (ANE+NaCl and NaCl), at both time points, but not in the ANE treatment (Fig 6). Saline conditions down-regulated ath-miRNA398, which targets two of the three copper/zinc superoxide dismutases of *Arabidopsis* (i.e. *AtCSD1* and *AtCSD2*) by triggering their cleavage, or by inhibiting translation of their mRNAs [49,50]. In this study, ANE was found to down-regulate ath-miRNA398b-5p and ath-miRNA398c but saline conditions had no clear inhibitory effects on these miRNAs. Interestingly, in spite of this trend, after 12 h *AtCSD1* was found to be down-regulated in plants treated with ANE in the presence of NaCl, while its expression was induced in the presence of NaCl and found to reach a maximum at 12 h (Fig 6).

DGE analysis revealed that the expression levels of miR399, miR827 and miR2111, all known to be involved in the regulation of phosphate uptake, were also influenced by the application of ANE. The expression of ath-miR399b, ath-miR399c-3p and ath-miR399c-5p was reduced by ANE and ANE+NaCl at 6 h and 12 h. NaCl alone enhanced the expression of these miRNA at both time points. Overall, the application of ANE, in the presence of NaCl, was associated with a lower expression of ath-miR399a, ath-miR399b, ath-miR399c-3p and ath-miR399c-5p as compared to plants treated with NaCl alone (Fig 6). *AtUBC24* (*PHO2)*, the well characterized target gene of ath-miR399b, -c, and–f [51], and predicted target of ath-miR399a, was also differentially regulated by the application of ANE (Fig 6). The treatment with ANE+NaCl resulted in a higher expression of *AtUBC24* at all time points, as compared to the NaCl alone treatment. However, the difference between these treatments was significant only after 12 h (Fig 6). In addition to *AtUBC24*, ath-miR399c also regulates the expression of *AtWAK2* (Fig 6). After 12 h, *AtWAK2* was significantly induced (2.0 fold) in the ANE+NaCl treatment, as compared to the NaCl (1.5 fold) but this effect cannot be attributed to ath-miR399c-3p and 5p as their expression was very similar over the time points (Fig 6). NGS showed that NaCl but not ANE or ANE+NaCl substantially induced the expression of miR827 which was accompanied by a moderate increase, at 12 h, in the expression of *AtNLA*, an ubiquitin E3 ligase which is involved in the degradation of PHT1 at the plasma membrane [52,53]. The application of ANE in the NaCl-treated plants marginally reduced the expression of *AtNLA* at 6 h while no effect was observed after 12 h of treatment (Fig 6). Ath-miR2111b targets *At3g27150*, a galactose oxidase/kelch repeat super-family protein which is induced in phosphate starvation conditions [54]. The application of ANE, in the presence of NaCl, limited the transient up-regulation trend of ath-miR2111b as observed in the NaCl treatment at 6 h (Fig 6). In the first time point *At3g27150* had a contrasting expression in salinity conditions, being up-regulated by NaCl and down-regulated by ANE+NaCl; however, at 12 h the expression pattern was almost similar in all treatments (Fig 6).

Ath-miR395 plays a key role in sulphate homeostasis by regulating sulphate uptake, transport and assimilation in *Arabidopsis thaliana* [49,55–57]. miR395 negatively regulated the expression of *AtSULTR2*;1 [56]. Applications of ANE had a strong influence in the expression of ath-miR395b,-c and -f while the effects of NaCl alone were more discrete (Fig 6). Saline conditions were found to strongly induce the expression of *AtSULTR2*;*1* however, the application of ANE in the presence of NaCl limited this up-regulation trend, and this effect was statistically significant at the earliest time point, i.e, after 6 h (Fig 6). Expression of ATP sulphurylase 1, 3 and 4 was very similar at 6 h in salinity conditions while at 12 h the up-regulation in ANE+NaCl treatments as compared to plants treated with NaCl was more pronounced (Fig 6).

### ANE improved growth and root system architecture of *Arabidopsis* in the presence of saline stress and phosphate starvation

ANE improved the growth and development of plants in the presence of NaCl. Phosphorus starvation and salinity severely reduced plant fresh and dry weight (S8 Fig). The application of ANE improved the fresh and dry weight and relative water content of those plants grown in phosphorus-deprived media supplemented with 100 mM NaCl (S8 Fig). An increase in the length of the primary root, number of lateral roots and the average length of lateral roots was found in ANE-treated plants in the presence of both, salinity and phosphate starvation, as compared to the control plants (S9 Fig).

## Discussion

Soil salinity is a major abiotic constraint, known to significantly affect global crop yields and productivity in a negative manner [1,58]. In order to meet the food requirements of an increasing global population, researchers are focusing on sustainable approaches such as the use of bioeffectors and biostimulants from natural sources in order to improve plant growth and productivity in changing and increasingly harsher environmental conditions. *Ascophyllum nodosum* extract (ANE) is a well-characterized bioeffector and biostimulant used for the improvement of plant growth and for the mitigation of various abiotic and biotic stresses in plants [25–28,31–33,59,60]. The ability of plants to survive in soils with elevated salinity depends on the plasticity of root architecture [61,62]. Stress signals and nutrient deficiencies are known to modulate the architecture of the root system by regulating the growth of the primary root and the development of lateral roots [62]. Osmotic stresses inhibited the emergence of lateral roots, while salinity stresses impeded the growth of the primary root through a decrease in cell division and elongation [62,63]. The *in vitro* assays performed in this study showed that ANE application improves both shoot and root biomass productivity of *Arabidopsis* in the presence of NaCl. ANE treatment reduced the harmful effects of salinity by enhancing the growth of primary and lateral roots. There is a general agreement that salinity stress modulates root system architecture by differentially regulating the distribution of auxins in roots, which in turn determines the direction of growth and the post embryonic development of lateral roots [64]. Rayorath et al. (2007) [59] showed that the application of ANE modulated the concentration and the localization of auxin within *Arabidopsis*. Thus, it is plausible that ANE improved root system architecture of the plants growing in the presence of NaCl by regulating the spatial distribution of auxin within the root zone.

Various studies have revealed components of the molecular mechanisms involved in ANE-mediated salinity tolerance in *Arabidopsis thaliana* [27,36]. Jones-Rhoades and Bartel (2004) [65] demonstrated the relationships between plant miRNA and stress responses. Aiming to understand better the mode of action of ANE in *Arabidopsis* growing in normal conditions and in the presence of salinity stress, we identified 106 miRNAs that were differentially regulated by ANE application. The exploration of the expression patterns of a number of validated or predicted targets of several miRNA in *Arabidopsis* provided further evidence of the modulatory effects of ANE on gene expression and uncovered some of the paths through which ANE might be able to mitigate salinity stress in plants. DGE analyses revealed that the expression of ath-miR842, ath-miR5645, ath-miR395, ath-miR8175 and ath-miR396 was higher in the presence of ANE+NaCl as compared to NaCl alone after 6 h. While that of ath-miR169 and ath-miR396a-5p was higher, in comparable conditions, after 12 h. Ath-miR399a, ath-miR399c-5p and ath-miR827 showed little induction or were repressed by ANE+NaCl after 6 h, as compared to control. The expression of these miRNAs in plants treated with NaCl alone were clearly up-regulated. After 12 h the miRNAs showed further reductions in their expression in the presence of ANE+NaCl, as compared to NaCl alone. This trend indicated that the addition of ANE limited the enhancing effects on gene expression of several miRNAs determined by salinity stress. Previous reports on the role of miRNA in relieving abiotic stresses identified several miRNAs that are stress-regulated [11,66]. Microarray analysis revealed that the treatment of Arabidopsis plants with 300 mM NaCl determined the up-regulation of ath-miR396, ath-miR169, ath-miR168, ath-miR167, ath-miR165, ath-miR319, ath-miR159 and ath-miR156 [66]. Not all these miRNAs were found to be induced, or strongly induced by salinity stress in this study likely due to the fact that the concentration of NaCl used was significantly lower, i.e. only 150 mM. Nevertheless, ANE in the presence 150 mM NaCl, modulated the expression of a number of miRNAs known to play versatile roles in the responses of plants to salinity stress.

By maneuvering the key components of a complex gene network, miRNAs are helping plants to mitigate salinity stress [1,10,67]. However, miRNAs do not directly regulate plant responses to environmental stresses [23]. After the biogenesis of mature miRNAs, they form a RNA-induced, silencing complex which binds to the mRNAs of the target genes in order to regulate their expression, either by adenylation, translational inhibition or by degradation of target mRNA [68,69]. To further understand the roles of ANE in salinity tolerance in *Arabidopsis*, the expression of 14 target genes of miRNAs, which were found to be modulated by salinity stress, was also studied. Ath-miR396 is conserved within different plant species and it is predicted to target *AtGRF7* in *Arabidopsis* [70]. Growth-regulating factors (GRF) constitute a family of transcription factors that are able to modulate organ growth (i.e. roots, shoots and leaves) under normal and stress conditions [15]. At 6 h after treatments, ANE in the presence of NaCl, but not NaCl alone, slightly induced the expression of ath-miR396a-5p, which down-regulated the expression of *AtGRF7* up to 12 h. In *Arabidopsis*, it has been shown that *AtGRF7* down-regulated the expression of *AtDREB2a* by binding to its promoter element which then acted as a down-regulator for salinity tolerance [47,70,71]. Indeed, compared to NaCl alone, lower levels of *AtGRF7* in the ANE+NaCl treatment led to the higher expression of *AtDREB2a* and *AtRD29*. It is likely that these differences contributed to the enhanced salinity tolerance observed in plants that received the ANE+NaCl treatments, as compared to the plants treated only with NaCl.

Earlier reports showed that salinity stress induced the expression of ath-miR169 in *Arabidopsis* [10,72,73], which is predicted to target transcription factors NFYA [48]. *AtNFYA1* functions in an ABA-dependent manner and regulated plant growth after germination in the presence of salinity stress [74]. In *Arabidopsis* and rice, under saline conditions, *NFYA1* was found to be up-regulated and its expression to be partially dependent on the down-regulation of miR169 [48,74]. Ath-miR169 family was also shown to play a key role in stress-induced flowering in plants [75]. In *Arabidopsis* the over-expression of ath-miRNA169 resulted in early flowering, while the over-expression of its target *AtNFYA2* resulted in late flowering [75]. The application of ANE, along with NaCl, determined a reduction in the expression of ath-miR169g-5p, 6 h after treatment but not after 12 h, while NaCl alone enhanced the levels of this miRNA at both time points. The expression of *AtNFYA1* and *AtNFYA2* was higher in the ANE+NaCl treatment, as compared to NaCl alone, and interestingly, this trend continued from 6 to 12 h even though at 12 h the transcript levels of ath-miR169g-5p were 3.2 times higher, as compared to the former time point. The delayed induction of ath-miR169g-5p in the ANE+NaCl treatment and the higher up-regulation of *AtNFYA1* by ANE+NaCl, as compared to NaCl, was consistent with the suggested mitigating effects of ANE under salinity stress and with a partial control of miR169 over *NFYA1* expression [48,74]. Similarly, the expression pattern of *AtNFYA2* suggested a very limited influence of miRNA169g-5p over its transcript abundance in saline conditions (ANE+NaCl and NaCl alone). Noteworthy, in our experiment, a decreased expression level of *AtNFYA2* was found in the ANE treatment while the ANE+NaCl and NaCl treatments were associated with an up-regulation trend. The expression pattern of *AtNFYA2* matched the flowering pattern observed later on in our experiment, i.e., after samples were collected for NGS and qPCR analyses: plants treated with ANE flowered more or less at the same time as controls, plants treated with ANE+NaCl flowered late while in the NaCl treatment inflorescence development and flowering were further delayed (Fig 1).

The exposure of plants to salinity stress has been reported to result in the accumulation of reactive oxygen species (ROS) in their cells [76,77]. Ath-miR398 is negatively regulated by salinity stress and it has been shown to down-regulate the expression of CSD1 and CSD2, two copper/zinc superoxide dismutase that act as ROS scavengers. Both enzymes are important for stress resistance and survival in plants [49,50,68]. Saline conditions had no clear inhibitory effects on ath-miRNA398b-5p and ath-miRNA398c. ANE and NaCl treatments had little effects on its expression while ANE+NaCl induced these miRNAs at both time points. If considering all treatments and time points *AtCSD1* expression was not negatively correlated with that of ath-miR398b-5p and ath-miRNA398c, thereby suggesting that these miRNAs only partially regulated the expression of superoxide dismutase. The decreased expression of *AtCSD1* at 12 h after the ANE+NaCl treatment was surprising as ANE and NaCl in separate treatments up-regulated this enzyme. It is likely that the elevated levels of ath-miRNA398b-5p and ath-miRNA398c, seen only in the ANE+NaCl treatments, over the two time points, were sufficient to negatively regulate transcript levels of *AtCSD1*. Clearly, additional studies, using more time points and possibly higher salinity are needed to further explore the significance of this finding.

Phosphorus, being a key component of nucleic acids, phospholipids, phosphoproteins, dinucleotides and adenosine triphosphate, is therefore necessary for several important physiological plant processes such as photosynthesis, energy storage and transfer and enzyme regulation. Drought and salinity cause nutritional imbalance in plants [4]. Plant exposure to salinity stress causes phosphorus starvation-like responses by impairing phosphate uptake due to the high ionic concentrations in the soil that reduce the absorption of phosphate by decreasing the solubility of Ca-P minerals [78]. Differential gene expression analysis revealed that the abundance of a few miRNAs that were involved in phosphate starvation regulation were influenced by the ANE+NaCl treatment. The results of this study suggested that ANE treatment influenced phosphate uptake in *Arabidopsis* under salinity stress by regulating the expression of miR399, which is known to be involved in phosphate homeostasis. Phosphorus starvation was reported to induce the expression of several miR399 (miR399b, miR399c and miR399f) which suppressed the accumulation of transcripts of their target gene *AtUBC24* (*PHO2)*, a ubiquitin conjugating E2 enzyme [51,79–81]. *AtUBC24* mediates the degradation of PHO1 and of PHT1 and, therefore, modulates phosphate uptake and root-to-shoot translocation [82,83]. The expression of ath-miR399a, ath-miR399b, ath-miR399c-3p and ath-miR399c-5p was either suppressed, or not strongly influenced, by ANE and by ANE+NaCl while the NaCl treatment clearly induced these miRNAs. Therefore, ANE reduced the up-regulation trend induced by saline conditions which resulted in an increased expression of the target genes *AtUBC24* and *AtWAK2*. Similarly, the up-regulation trend of ath-miR827 observed in the presence of NaCl, was limited in ANE+NaCl, which led to the higher expression of its target gene *AtNLA*, an ubiquitin E3 ligase with RING and SPX domains which also mediated the degradation of the phosphate co-transporter PHT1. The modulatory effects of ANE and ANE+NaCl on the expression of ath-miR399a, ath-miR399b, ath-miR399c-3p, ath-miR399c-5p and ath-miR2111b-3p indicated that the extract had the potential to influence phosphate homeostasis. Interestingly, we did not observe a clear negative correlation between the expression of these miRNA and their targets. This suggested that these miRNAs exerted only a partial control over their targets, or that they represented only one of the many regulatory routes of control. The lack of a temporal negative correlation between miRNAs and their targets has been reported in several other studies [84–86], including between ath-miR2111-5p and At3g27150 [54] and have been reviewed by Voinnet (2009) [87]. Nevertheless, the complexity of the different miRNA regulatory circuits involved in phosphate homeostasis were more than apparent when comparing the regulation trend observed for *AtUBC24* and *AtNLA*, which encodes for proteins related to ubiquitination processes that lead to the degradation of PHO1 and PHT1 which are essential in phosphate uptake and transfer within plants. While the expression of *AtUBC24* was increased in the presence of ANE, ANE+NaCl and NaCl, suggesting a possible reduction in the efficiency of Pi uptake and translocation, the expression of *AtNLA* was decreased in all three treatments, indicating a rather opposite trend in phosphate regulation, at least at the first time point. Also, in saline conditions, ANE enhanced the up-regulation trend of *AtUBC24* as compared to NaCl, while in the same conditions *AtNLA* expression was decreased. More studies, including the characterization of the expression of genes downstream of the targets of miRNAs are needed to clarify these effects. To further investigate the effects of ANE on phosphate homeostasis and in relieving phosphate starvation in plants exposed to salinity, the effect of ANE on growth and root system architecture (RSA) was also explored in *in vitro* experiments. ANE treatment was found to significantly improve growth, in terms of fresh and dry weight, as well as the RSA of *Arabidopsis* both in the presence of salinity, as well as the combination of phosphate starvation and salt stress. Under phosphate starvation, a strong response was observed in RSA, which resulted in a shift from main root growth to lateral root growth, this led to shorter roots, with numerous lateral roots, features that were also previously reported [61,62,88]. During phosphate stress, plants show an increased growth of the lateral roots with a higher level of branching [88]. However, application of ANE, in the phosphate-starved medium, resulted in a RSA with reduced lateral root growth and branching. This suggested that ANE applications to *Arabidopsis thaliana* improved phosphate homeostasis in the plants which were experimentally grown in phosphorus deprived media. The growth of primary and lateral roots was affected more severely in those plants which were exposed to a combination of phosphate starvation and salinity stress, rather than by either one of these stresses alone [61]. The application of ANE in phosphate-starved media resulted in reduced growth of the main root axis, whilst in the salinity and phosphorus starvation combination, the application of ANE resulted in significant increases in the length of both primary and lateral roots. A plausible hypothesis for this phenomenon is that ANE application protected the root tip and its meristematic cells from the detrimental effect of both stresses by modulating gene expression that led to improved re-location of the existing phosphate resources. This hypothesis was further supported by the fact that, based on our estimates, a concentration of 0.01% ANE represented a negligible source of phosphate, i.e. around 100 times less than the amount of 625 μM potassium phosphate that is present in the ½ MS medium [38]. Nevertheless, the *in vitro* experimental model employed an artificial medium and plants used in experiments were in their early developmental stage. Clearly, the hypothesis remains to be further validated using plants grown *in vivo*, either on a solid substratum or in hydroponics, conditions that would allow full development of plant.

Sulphur plays an important role in plant growth and development [56,85]. Cysteine is the precursor for most organic S compounds in plants and it is important for the biosynthesis of reduced glutathione (GSH), the main storage and transport form of reduced sulphur [55]. It is known that GSH plays an important role in abiotic stress tolerance by detoxifying ROS and is also involved in redox buffering [55]. In *Arabidopsis*, ath-miR395 targets two families of genes, ATP sulphurylases (*AtAPS*) and sulphate transporter 2;1 (*AtSULTR 2;1*). In the presence of a S-deficiency, ath-miR395 is up-regulated which suppresses the expression of *AtSULTR 2;1*, which then restricts the transport of sulphate from mature to young leaves [56]. Interestingly, in the current study, ath-miR395b, -c and–f were found to be up-regulated more by the application of ANE and of ANE in saline conditions than that of NaCl. ANE reduced the expression of *AtSULTR 2; 1*, while NaCl had the opposite effect. ANE application in salinity conditions balanced almost perfectly these opposed trends, clearly indicating a role of ANE in sulphur uptake, under normal and saline conditions. The transcripts of *AtAPS1*, *AtAPS3* and *AtAPS4* were also differentially regulated by all three treatments with the most obvious effects in the ANE treatment, in the presence of NaCl. Taken together, these results suggested that ANE applications may improve sulphur homeostasis in salinity treatments by modulating the expression of ath-miR395 and, in salinity conditions, by up-regulating the expression of its target genes.

In conclusion, the bioactive components of a commercial *A*. *nodosum* extract improved salinity stress tolerance in *Arabidopsis thaliana* by modulating the expression of different miRNAs through the priming of an extensive network of pathways, some of them related to nutrient uptake and their *in planta* transfer. This study, using *Arabidopsis* as the model organism, provides a holistic, fundamental explanation of the molecular mechanisms through which ANE improves salinity tolerance in plants. Treatments of ANE alone and ANE in salinity conditions modulated the expression of a plethora of miRNAs involved in essential regulatory circuits and of several genes that are direct targets of miRNAs. The magnitude of changes in the expression of several miRNAs caused by NaCl was reduced by an ANE treatment thereby suggesting an important role for this extract in mitigating salt tolerance in plants. This study provides the basis for applications of ANE as an important management tool which can enable important plant crops to better tolerate ever-increasing exposure to salt stress conditions in an environmentally friendly, positive way.

## Supporting information

S1 Fig Three-week-old *Arabidopsis* plants before being supplemented with water (a), 100 mM NaCl (b), 0.1% ANE (c), and 0.1% ANE with 100 mM NaCl (d). (TIF)

S2 Fig ANE improves growth of *Arabidopsis* in the presence of salinity. The effects of ANE in absence and presence of salinity stress on the in vitro growth of 7 days old *Arabidopsis* seedlings grown in ½ MS media **(a)** or supplemented with 100 mM NaCl **(b)**, 0.1% ANE **(c)**, and 0.1% ANE with 100 mM NaCl **(d)**. The effect of ANE on the fresh **(e)** and dry weight **(f)** and relative water content **(g)** of *Arabidopsis* seedlings from control and the three treatments. The effect of ANE on the three growth parameters was analyzed using ANOVA followed by Tukey. Means that do not share the same letter are significantly different (p<0.05). Error bars represents SE.
(TIF)

S3 Fig ANE modulates root system architecture of *Arabidopsis* in the presence of salinity. The effects of ANE in absence and presence of salinity stress on root system architecture of *Arabidopsis* seedlings grown on in vitro conditions. **(a)** The effect of ANE on the primary root length, **(b)** average length and **(c)** the number of lateral roots of *Arabidopsis* seedlings from control and the three treatments (ANE, ANE+NaCl and NaCl). Error bars represent SE.
(TIF)

S4 Fig **(a)** Average number of reads (in millions) obtained from sequencing the libraries from control and the 3 treatments (ANE, ANE+NaCl and NaCl) after 6 and 12 h of treatment. **(b)** Average number of miRNA mapped to the Arabidopsis genome in the libraries from control and the three treatments. Error bars represent SE.
(TIF)

S5 Fig Size distribution of miRNA sequences in the libraries from control and the three treatments (ANE, ANE+NaCl and NaCl), constructed from the leaves of *Arabidopsis* after 6 h (a) and 12 h (b) of treatment. (TIF)

S6 Fig MA plot of distribution of differentially expressed genes in *Arabidopsis* between the various treatments at 6 h. C: Control, T_1_: ANE, T_2_: ANE+NaCl, T_3_: NaCl.
(TIF)

S7 Fig MA plot of distribution of differentially expressed genes in *Arabidopsis* between the various treatments at 12 h. C: Control, T_1_: ANE, T_2_: ANE+NaCl, T_3_: NaCl.
(TIF)

S8 Fig The effects of ANE in absence and presence of salinity stress and of phosphate on the in vitro improves the growth of 7 days old *Arabidopsis* seedlings supplemented with 0 mM NaCl **(a)**, 0.01% ANE **(b)**, 100 mM NaCl **(c)**, and 0.01% ANE with 100 mM NaCl **(d)** and phosphate starved media with 0 mM NaCl **(e)**, 0.01% ANE **(f)**, 100 mM NaCl **(g)**, and 0.01% ANE with 100 mM NaCl **(h)**. The effect of ANE on the fresh and dry weight **(i, j)** and relative water content **(k)** of *Arabidopsis* seedlings growing with 0 and 100 mM NaCl in the ½ MS media and ½ MS media starved with phosphate. The effects of ANE and of phosphate starvation on the three growth parameters were analyzed using ANOVA followed by Tukey. Means that do not share the same letter are significantly different (p<0.05). Error bars represent SE.
(TIF)

S9 Fig The effects of ANE in absence and presence of salinity stress and of phosphate on the root system architecture of *Arabidopsis* seedlings in the presence of controls and phosphate-depleted medium ANE, ANE + 100 mM NaCl and 100 mM NaCl. The effect of ANE on the primary root length (a), average length (b), and number (c) of lateral roots of Arabidopsis seedlings. Error bars represent SE.
(TIF)

S1 Table List of the primers used in target gene expression analysis. (DOCX)

S2 Table Na^+^, K^+^, K^+^/Na^+^ ratio and P content of the *Arabidopsis* grown in the presence of control, ANE (T_1_), ANE+NaCl (T_2_) and NaCl (T_3_). (DOCX)

S3 Table Differential expression of conserved *Arabidopsis* miRNAs among control (C), ANE (T_1_) and ANE+NaCl (T_2_) and NaCl (T_3_) treatments at 6 h. (DOCX)

S4 Table The differential expression of conserved *Arabidopsis* miRNAs among control (C), ANE (T_1_) and ANE+NaCl (T_2_) and NaCl (T_3_) treatments at 12 h. (DOCX)

S5 Table List of target genes predicted. (DOCX)
