# Supplementary figures and images for: Ascophyllum nodosum extract mitigates salinity stress in Arabidopsis thaliana by modulating the expression of miRNA involved in stress tolerance and nutrient acquisition
Source: PLoS One. 2018 Oct 29;13(10):e0206221. doi: 10.1371/journal.pone.0206221 (PMC6205635; doi:10.1371/journal.pone.0206221)

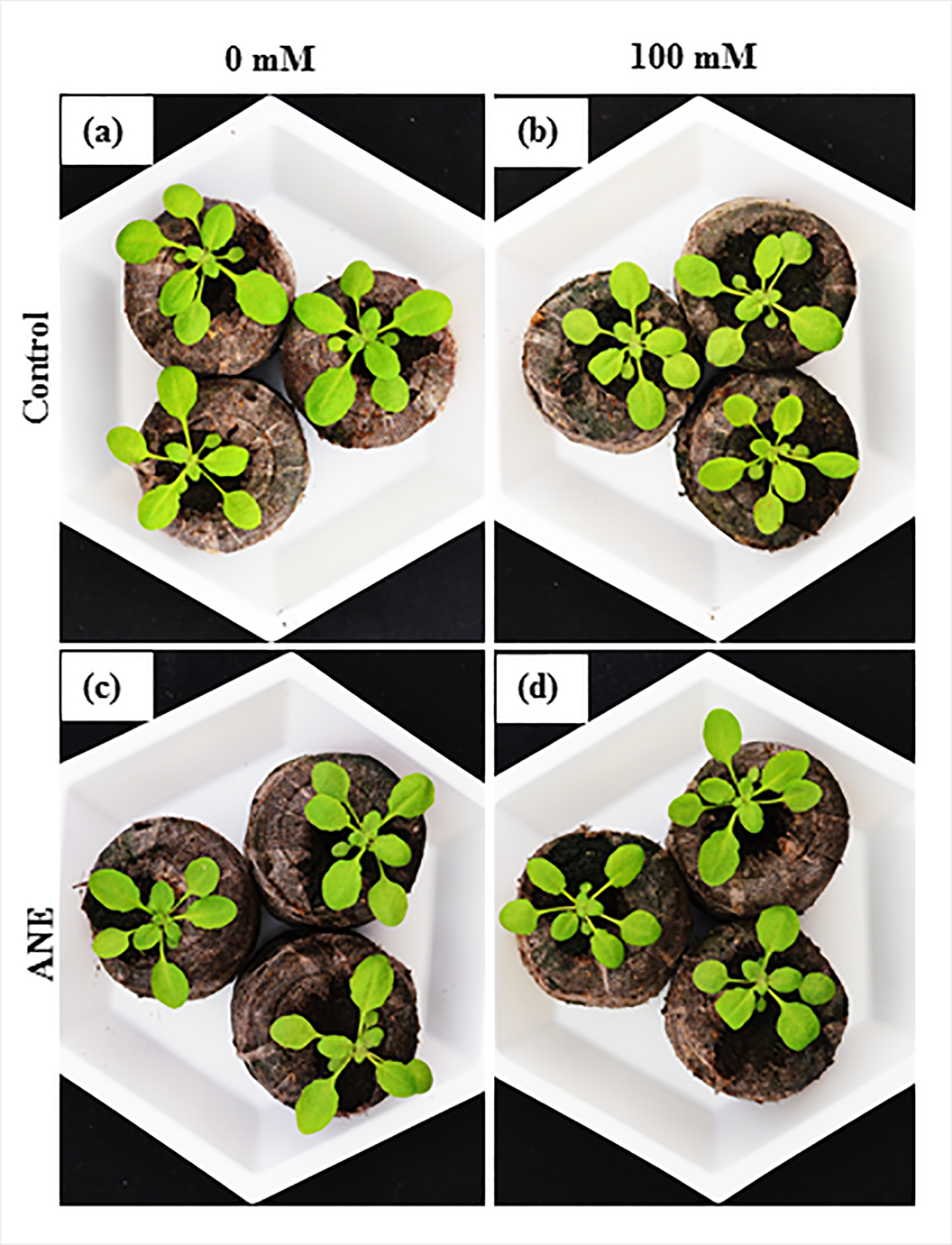

Supplement: S1 Fig — (TIF) [file pone.0206221.s001.TIF]

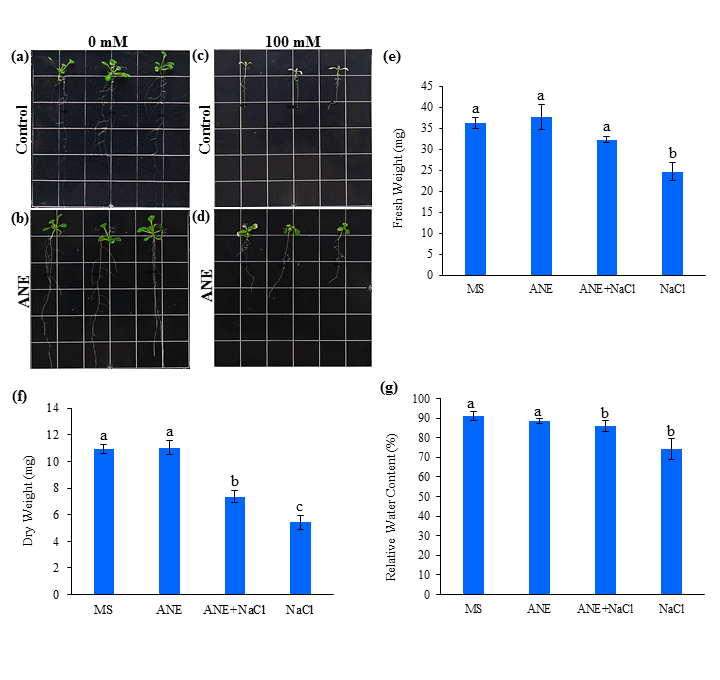

Supplement: S2 Fig — The effects of ANE in absence and presence of salinity stress on the in vitro growth of 7 days old Arabidopsis seedlings grown in ½ MS media (a) or supplemented with 100 mM NaCl (b), 0.1% ANE (c), and 0.1% ANE with 100 mM NaCl (d). The effect of ANE on the fresh (e) and dry weight (f) and relative water content (g) of Arabidopsis seedlings from control and the three treatments. The effect of ANE on the three growth parameters was analyzed using ANOVA followed by Tukey. Means that do not share the same letter are significantly different (p<0.05). Error bars represents SE. (TIF) [file pone.0206221.s002.TIF]

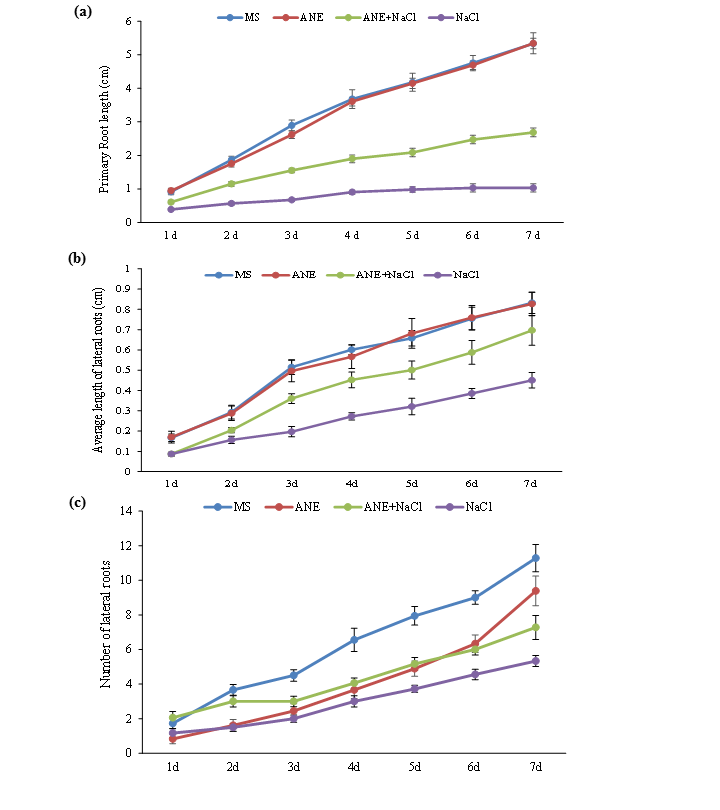

Supplement: S3 Fig — The effects of ANE in absence and presence of salinity stress on root system architecture of Arabidopsis seedlings grown on in vitro conditions. (a) The effect of ANE on the primary root length, (b) average length and (c) the number of lateral roots of Arabidopsis seedlings from control and the three treatments (ANE, ANE+NaCl and NaCl). Error bars represent SE. (TIF) [file pone.0206221.s003.TIF]

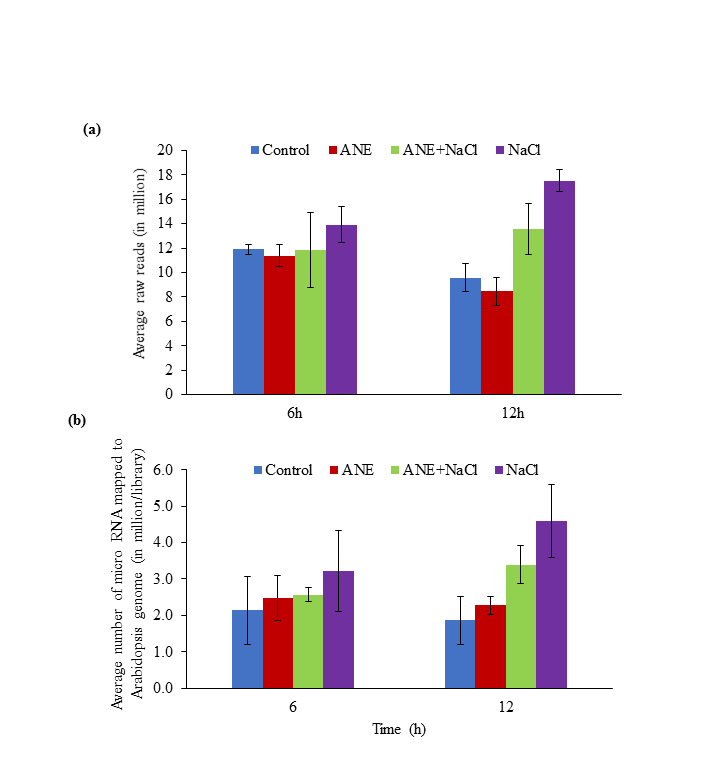

Supplement: S4 Fig — (a) Average number of reads (in millions) obtained from sequencing the libraries from control and the 3 treatments (ANE, ANE+NaCl and NaCl) after 6 and 12 h of treatment. (b) Average number of miRNA mapped to the Arabidopsis genome in the libraries from control and the three treatments. Error bars represent SE. (TIF) [file pone.0206221.s004.TIF]

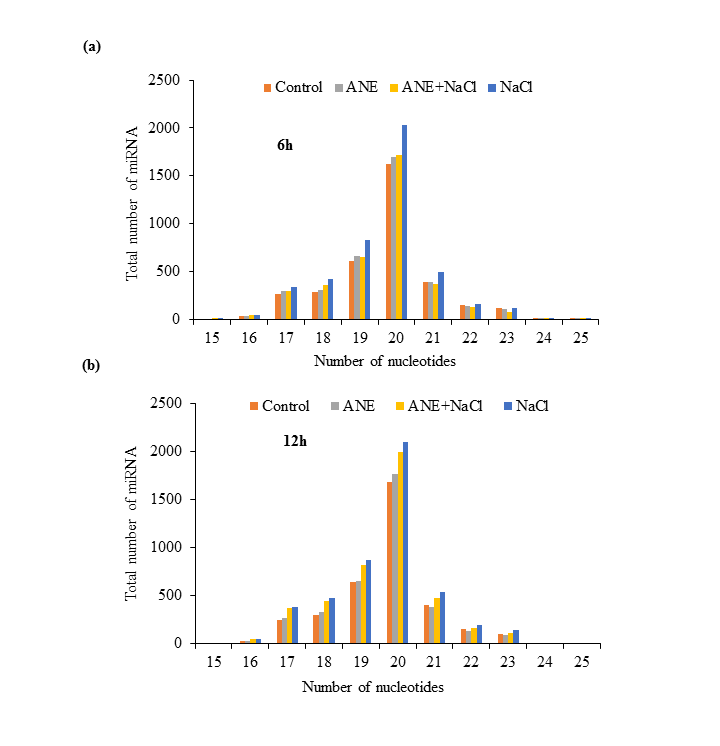

Supplement: S5 Fig — (TIF) [file pone.0206221.s005.TIF]

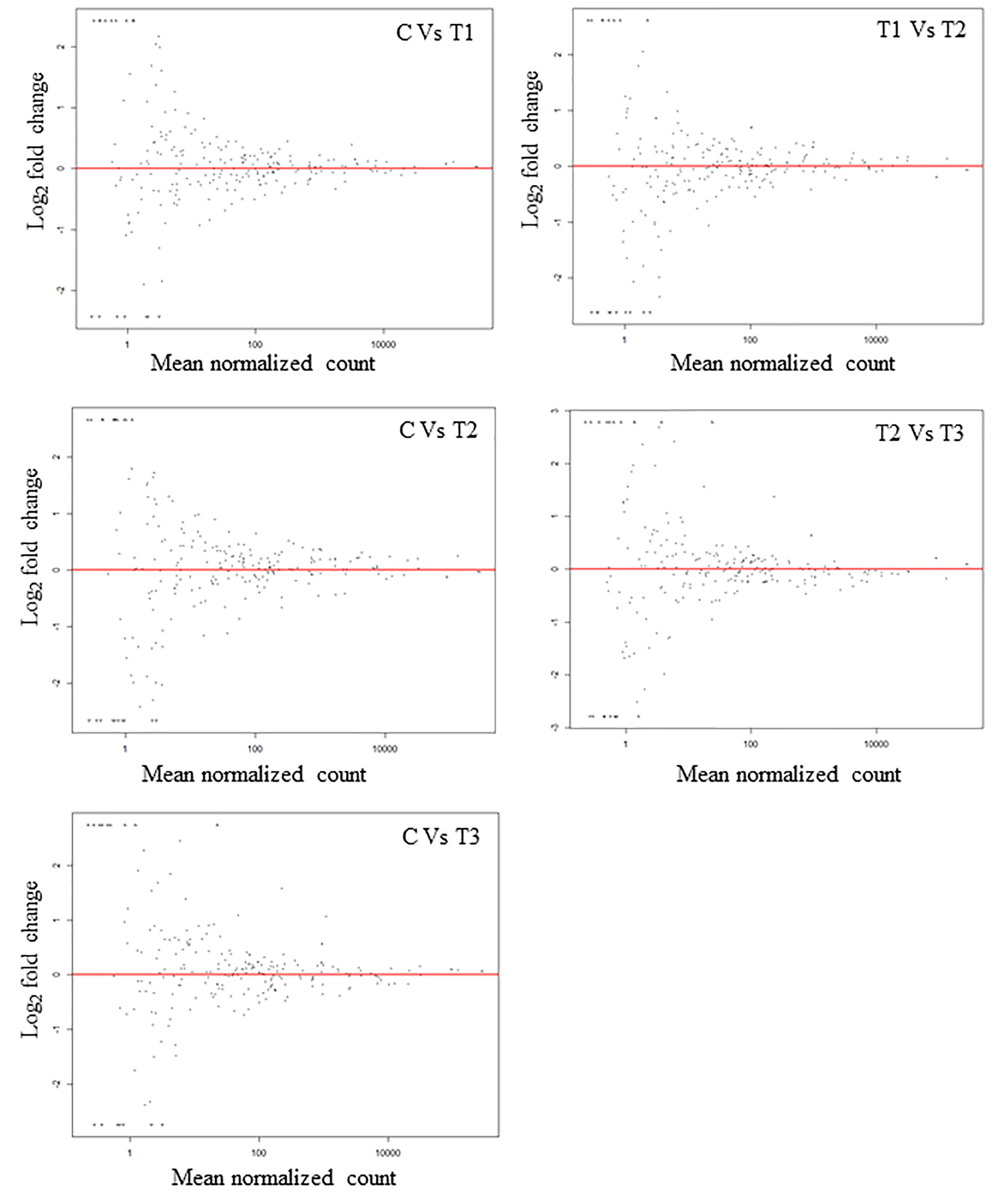

Supplement: S6 Fig — C: Control, T1: ANE, T2: ANE+NaCl, T3: NaCl. (TIF) [file pone.0206221.s006.TIF]

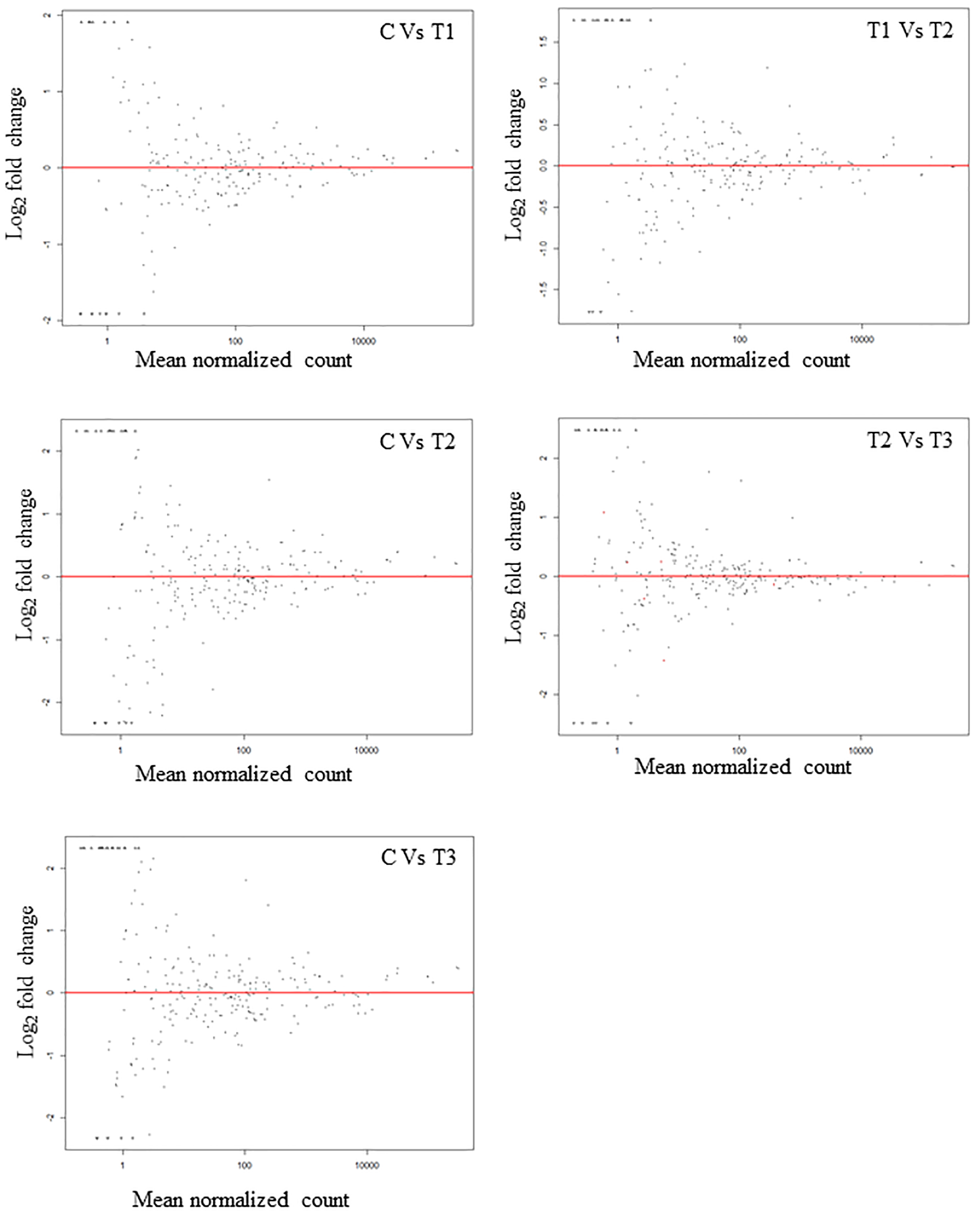

Supplement: S7 Fig — C: Control, T1: ANE, T2: ANE+NaCl, T3: NaCl. (TIF) [file pone.0206221.s007.TIF]

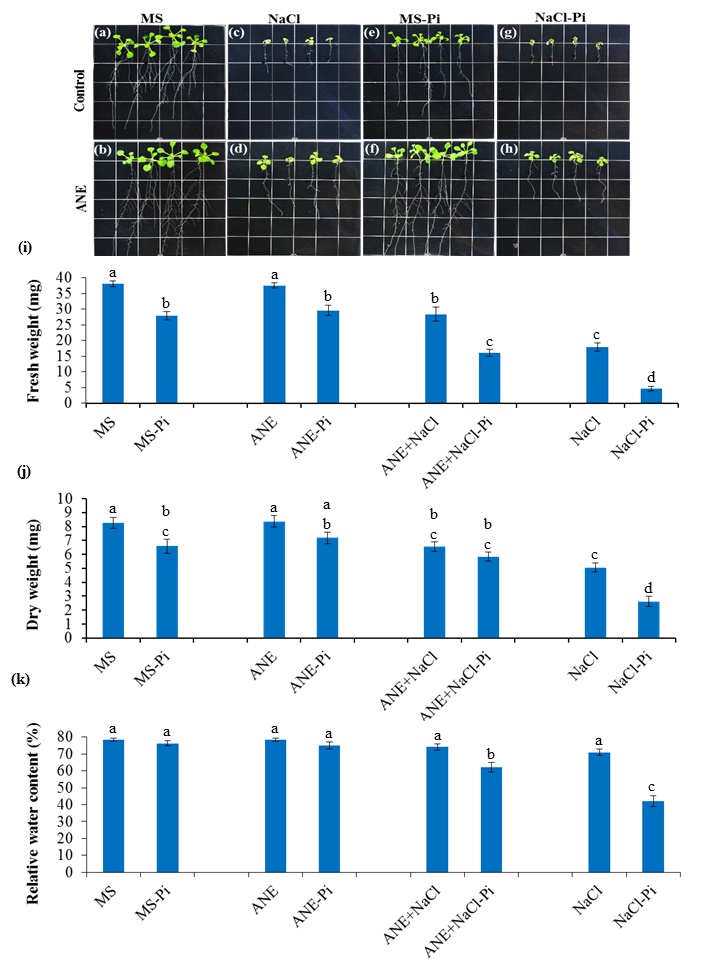

Supplement: S8 Fig — The effects of ANE in absence and presence of salinity stress and of phosphate on the in vitro improves the growth of 7 days old Arabidopsis seedlings supplemented with 0 mM NaCl (a), 0.01% ANE (b), 100 mM NaCl (c), and 0.01% ANE with 100 mM NaCl (d) and phosphate starved media with 0 mM NaCl (e), 0.01% ANE (f), 100 mM NaCl (g), and 0.01% ANE with 100 mM NaCl (h). The effect of ANE on the fresh and dry weight (i, j) and relative water content (k) of Arabidopsis seedlings growing with 0 and 100 mM NaCl in the ½ MS media and ½ MS media starved with phosphate. The effects of ANE and of phosphate starvation on the three growth parameters were analyzed using ANOVA followed by Tukey. Means that do not share the same letter are significantly different (p<0.05). Error bars represent SE. (TIF) [file pone.0206221.s008.TIF]

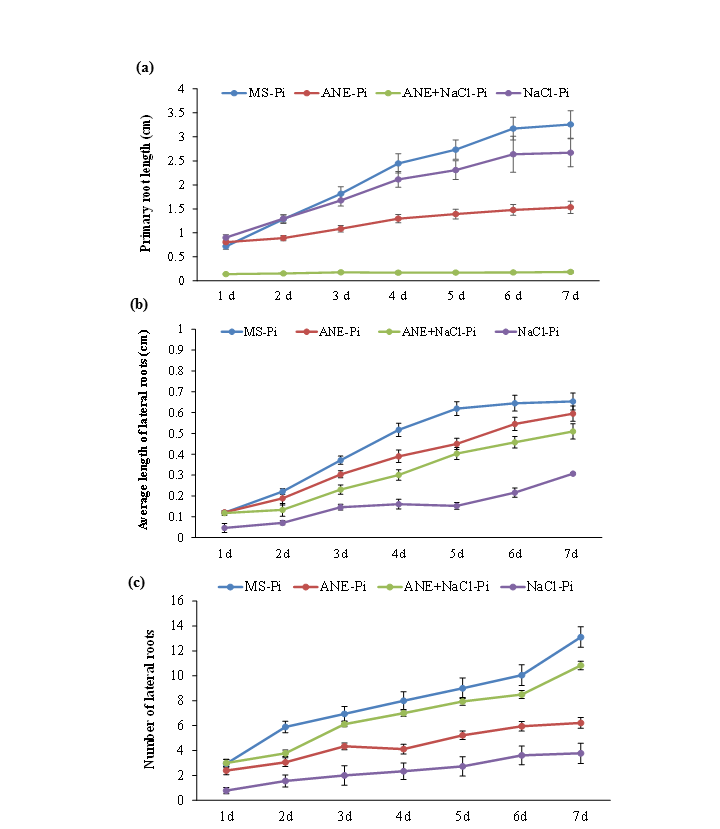

Supplement: S9 Fig — The effect of ANE on the primary root length (a), average length (b), and number (c) of lateral roots of Arabidopsis seedlings. Error bars represent SE. (TIF) [file pone.0206221.s009.TIF]
