# Supplementary material for: Ascophyllum nodosum extract mitigates salinity stress in Arabidopsis thaliana by modulating the expression of miRNA involved in stress tolerance and nutrient acquisition
Source: PLoS One. 2018 Oct 29;13(10):e0206221. doi: 10.1371/journal.pone.0206221 (PMC6205635; doi:10.1371/journal.pone.0206221)
Supplement: S1 Table — (DOCX) [file pone.0206221.s010.docx]

**S1 Table. List of the primers used in target gene expression analysis.**

|  | **Target Gene/Acession No** | **Sequence** | **Size** |
| --- | --- | --- | --- |
| 1. | ATP sulfurylase1 (*AtAPS1*) | 5’- GCTCCGTCGTTAACATGTCC -3’  5’- CCGTCGTACCCCATGTTCTA-3’ | **186** |
| 2. | ATP sulfurylase3 (*AtAPS3*) | 5’-GCGGCGGATTTGCCGAGAGT-3’  5’-CCCACGAAGAGGACTAGCCCAAC-3’ | **90** |
| 3. | ATP sulfurylase4 (*AtAPS4*) | 5’-AGCGAAGGCTGGGCAAGTCC-3’  5’- AGCCGTCTTCGAGCCGGAAC-3’ | **94** |
| 4. | Sulphate transporter 2;1 (*AtSULTR 2;1*) | F-5’- TTTTGAATCTCTCTCACATCAAGTTCTCCC-3’  R-5’- TGGTCTTGACCGGCTTGTGCG-3’ | **114** |
| 5. | *AtCSD1* | F-5’- CCCCGATGGTAAAACACACG-3’  5’- TGAGTTCATGGCCTCCCTTT-3’ | **206** |
| 6. | *AtGRF7* | F-5’-TTAGCGGAGATACTGAGGCC-3’  R-5’-ACTCCTAAACCTGGCTGCTT-3’ | **157** |
| 7. | *AtDREB2a* | F- TTGGCTGAGCGAGTTTGAAC  R- CGGTCCTGATTTAAGCCTGC | **243** |
| 8. | *AtRD29A* | F- AGGAAGAGTCGGCTGTTTCA  R- TGCTTCTCGTCGACAAGTCT | **189** |
| 9. | *AtWAK2* | F-TCCAGATCTAAACCCGCCAA  R-GAAACAGGAGACGACGAGGA | **234** |
| 10. | *AtUBC24/AtPHO2* | F-GTCTACGTCGATTGGGTTGC  R-TGAGATTGTAGGCAGCCACA | **208** |
| 11. | *AtNFYA1* | F-ATGACCCAGCGCTCTCTATC  R-GTCAAGTGGCAGAGCTGTTC | **203** |
| 12. | *AtNFYA2* | F- TCATTCGCCGGAGTTAAGGT  R- TCGGTGGCTGAGAAAATCCA | **190** |
| 13. | *AtNLA* | F-GACTTTCGACGATGGGAAGC  R-TGCGGTTTTCAAGCCATCAA | **193** |
| 14. | *At3g27150* | F-AAATGGTTCAAAGGTCCCGC  R-CACCCAGAGCAGAATTTCCG | **209** |
| 15. | *AtLEA14* | F-GGACTTCGTGGCGGATAAAC  R-TCAAGAGCCGTCATGTCCTT | **246** |
| 16. | *AtLAC2* | F-CACTAGCTACGCCGCAAATT  R-TTGGTCCTTGGCATGTTTGG | **155** |
| 17. | *AtHD* | F-GAAGCGGCACAAGAAGAGAG  R-GGCCGGGGTTTTGTTCATAT | **167** |
