# Supplementary material for: Ascophyllum nodosum extract mitigates salinity stress in Arabidopsis thaliana by modulating the expression of miRNA involved in stress tolerance and nutrient acquisition
Source: PLoS One. 2018 Oct 29;13(10):e0206221. doi: 10.1371/journal.pone.0206221 (PMC6205635; doi:10.1371/journal.pone.0206221)
Supplement: S2 Table — (DOCX) [file pone.0206221.s011.docx]

**S2 Table. Na^+^, K^+^, K^+^/Na^+^ ratio and P content of the Arabidopsis grown in the presence of control, ANE (T_1_), ANE+NaCl (T_2_) and NaCl (T_3_).**

| **Treatments** | **Na^+^ content (mmol g^-1^ DW)** | **K^+^ content (mmol g^-1^ DW)** | **K^+^/Na^+^ ratio** | **P content (µmol g^-1^ DW)** |
| --- | --- | --- | --- | --- |
| Control | 00.075 ± 0.01^c^ | 00.634 ± 0.09^b^ | 08.361 ± 0.490^b^ | 211.41±22.71^ab^ |
| T_1_ | 00.121 ± 0.01^c^ | 01.419 ± 0.11^a^ | 11.936 ± 0..649^a^ | 279.40±23.57^a^ |
| T_2_ | 10.904 ± 0.25^b^ | 01.300 ± 0.09^a^ | 00.119 ± 0.006^c^ | 180.59±07.27^b^ |
| T_3_ | 19.087 ± 1.39^a^ | 01.164 ± 0.03^a^ | 00.062 ± 0.004^c^ | 141.31±07.02^b^ |
