# Supplementary material for: Ascophyllum nodosum extract mitigates salinity stress in Arabidopsis thaliana by modulating the expression of miRNA involved in stress tolerance and nutrient acquisition
Source: PLoS One. 2018 Oct 29;13(10):e0206221. doi: 10.1371/journal.pone.0206221 (PMC6205635; doi:10.1371/journal.pone.0206221)
Supplement: S3 Table — (DOCX) [file pone.0206221.s012.docx]

|  | **S3 Table. Differential expression of conserved *Arabidopsis* miRNAs among control (C), ANE (T_1_) and ANE+NaCl (T_2_) and NaCl (T_3_) treatments at 6 h.** | | | | | | | | | | | | |
| --- | --- | --- | --- | --- | --- | --- | --- | --- | --- | --- | --- | --- | --- |
| S.No. | miRNA family | Average number of reads per library | | | | Fold Change | | | | Significant change | | | |
|  |  | Control | ANE  (T_1_) | ANE+  NaCl (T_2_) | NaCl (T_3_) | T_1_ Vs C | T_2_ Vs C | T_3_ Vs C | T_2_ Vs T_3_ | T_1_ Vs C | T_2_ Vs C | T_3_ Vs C | T_2_ Vs T_3_ |
| 1. | ath-miR156g | 2 | 3 | 3 | 4 | 1.16 | 1.08 | 1.42 | 0.76 |  |  |  |  |
| 2. | ath-miR156h | 8 | 8 | 10 | 12 | 1.04 | 1.31 | 1.53 | 0.86 |  |  |  |  |
| 3. | ath-miR156j | 12 | 15 | 19 | 24 | 1.26 | 1.61 | 2.01 | 0.80 |  |  | ** |  |
| 4. | ath-miR157a-3p | 35 | 36 | 31 | 40 | 1.02 | 0.88 | 1.14 | 0.77 |  |  |  | * |
| 5. | ath-miR157a-5p | 3195 | 3025 | 3065 | 2873 | 0.95 | 0.96 | 0.90 | 1.07 |  |  |  |  |
| 6. | ath-miR157b-3p | 35 | 36 | 31 | 40 | 1.02 | 0.88 | 1.14 | 0.77 |  |  |  | * |
| 7. | ath-miR157b-5p | 3195 | 3025 | 3065 | 2873 | 0.95 | 0.96 | 0.90 | 1.07 |  |  |  |  |
| 8. | ath-miR157c-3p | 273 | 277 | 270 | 257 | 1.01 | 0.99 | 0.94 | 1.05 |  |  |  |  |
| 9. | ath-miR157c-5p | 4343 | 4036 | 3921 | 3687 | 0.93 | 0.90 | 0.85 | 1.06 |  |  |  |  |
| 10. | ath-miR157d | 278 | 269 | 307 | 254 | 0.97 | 1.10 | 0.91 | 1.21 |  |  |  |  |
| 11. | ath-miR158a-5p | 61 | 62 | 49 | 49 | 1.02 | 0.81 | 0.80 | 1.01 |  |  |  |  |
| 12. | ath-miR158b | 130 | 146 | 180 | 148 | 1.12 | 1.38 | 1.14 | 1.21 |  |  |  |  |
| 13. | ath-miR159a | 11743 | 11707 | 13631 | 12600 | 1.00 | 1.16 | 1.07 | 1.08 |  |  |  |  |
| 14. | ath-miR159c | 178 | 147 | 159 | 165 | 0.82 | 0.89 | 0.92 | 0.96 |  |  |  |  |
| 15. | ath-miR160c-3p | 16 | 17 | 19 | 16 | 1.08 | 1.19 | 0.99 | 1.20 |  |  |  |  |
| 16. | ath-miR164c-3p | 2 | 2 | 2 | 3 | 1.22 | 1.31 | 1.58 | 0.83 |  |  |  |  |
| 17. | ath-miR165a-3p | 36812 | 37713 | 34139 | 37887 | 1.02 | 0.93 | 1.03 | 0.90 |  |  |  |  |
| 18. | ath-miR165b | 35918 | 36878 | 33330 | 36960 | 1.03 | 0.93 | 1.03 | 0.90 |  |  |  |  |
| 19. | ath-miR166a-3p | 114097 | 113666 | 112504 | 114203 | 1.00 | 0.99 | 1.00 | 0.99 |  |  |  |  |
| 20. | ath-miR166b-3p | 106830 | 106903 | 105476 | 107185 | 1.00 | 0.99 | 1.00 | 0.98 |  |  |  |  |
| 21. | ath-miR166c | 106899 | 106958 | 105522 | 107235 | 1.00 | 0.99 | 1.00 | 0.98 |  |  |  |  |
| 22. | ath-miR166d | 106894 | 106954 | 105519 | 107231 | 1.00 | 0.99 | 1.00 | 0.98 |  |  |  |  |
| 23. | ath-miR166e-3p | 106794 | 106862 | 105435 | 107142 | 1.00 | 0.99 | 1.00 | 0.98 |  |  |  |  |
| 24. | ath-miR166f | 106794 | 106862 | 105435 | 107143 | 1.00 | 0.99 | 1.00 | 0.98 |  |  |  |  |
| 25. | ath-miR166g | 106860 | 106914 | 105477 | 107189 | 1.00 | 0.99 | 1.00 | 0.98 |  |  |  |  |
| 26. | ath-miR167a-3p | 2141 | 2262 | 2526 | 2091 | 1.06 | 1.18 | 0.98 | 1.21 |  |  |  |  |
| 27. | ath-miR167d | 1074 | 903 | 812 | 865 | 0.84 | 0.76 | 0.81 | 0.94 | * | * |  |  |
| 28. | ath-miR168a-3p | 1092 | 1388 | 1217 | 897 | 1.27 | 1.11 | 0.82 | 1.36 |  |  |  |  |
| 29. | ath-miR168b-3p | 57 | 51 | 66 | 65 | 0.90 | 1.17 | 1.14 | 1.02 |  |  |  |  |
| 30. | ath-miR169a-3p | 31 | 35 | 37 | 33 | 1.13 | 1.21 | 1.08 | 1.12 |  |  |  |  |
| 31. | ath-miR169f-3p | 47 | 55 | 39 | 32 | 1.16 | 0.82 | 0.68 | 1.21 |  |  |  |  |
| 32. | ath-miR169g-5p | 3 | 3 | 2 | 4 | 1.01 | 0.75 | 1.48 | 0.51 |  |  |  |  |
| 33. | ath-miR169h | 75 | 71 | 74 | 61 | 0.94 | 0.99 | 0.80 | 1.23 |  |  |  |  |
| 34. | ath-miR169i | 77 | 73 | 75 | 62 | 0.95 | 0.98 | 0.80 | 1.22 |  |  |  |  |
| 35. | ath-miR169k | 75 | 71 | 74 | 61 | 0.94 | 0.99 | 0.80 | 1.23 |  |  |  |  |
| 36. | ath-miR169m | 78 | 73 | 75 | 62 | 0.93 | 0.96 | 0.79 | 1.21 |  |  |  |  |
| 37. | ath-miR171a-5p | 39 | 38 | 38 | 40 | 0.97 | 0.95 | 1.01 | 0.94 |  |  |  |  |
| 38. | ath-miR171b-5p | 17 | 22 | 20 | 17 | 1.33 | 1.20 | 1.01 | 1.19 | * |  |  |  |
| 39. | ath-miR171c-5p | 13 | 17 | 16 | 16 | 1.32 | 1.23 | 1.26 | 0.98 | * |  |  |  |
| 40. | ath-miR173-5p | 26 | 23 | 24 | 30 | 0.88 | 0.92 | 1.13 | 0.82 |  |  |  |  |
| 41. | ath-miR1888a | 5 | 8 | 7 | 8 | 1.72 | 1.52 | 1.85 | 0.82 | * |  | * |  |
| 42. | ath-miR2111b-3p | 2 | 1 | 3 | 4 | 0.65 | 1.72 | 2.57 | 0.67 |  |  | * |  |
| 43. | ath-miR2933a | 3 | 2 | 4 | 3 | 0.79 | 1.40 | 1.10 | 1.27 |  |  |  |  |
| 44. | ath-miR391-3p | 147 | 150 | 150 | 163 | 1.02 | 1.02 | 1.11 | 0.92 |  |  |  |  |
| 45. | ath-miR393a-5p | 286 | 235 | 245 | 242 | 0.82 | 0.86 | 0.85 | 1.01 |  |  |  |  |
| 46. | ath-miR393b-5p | 286 | 235 | 245 | 242 | 0.82 | 0.86 | 0.85 | 1.01 |  |  |  |  |
| 47. | ath-miR395b | 7 | 9 | 12 | 6 | 1.26 | 1.85 | 0.91 | 2.03 |  | * |  | * |
| 48. | ath-miR395c | 7 | 9 | 12 | 6 | 1.26 | 1.85 | 0.91 | 2.03 |  | * |  | * |
| 49. | ath-miR395f | 7 | 9 | 12 | 6 | 1.26 | 1.85 | 0.91 | 2.03 |  | * |  | * |
| 50. | ath-miR396a-5p | 2895 | 3078 | 3068 | 2514 | 1.06 | 1.06 | 0.87 | 1.22 |  |  |  |  |
| 51. | ath-miR396b-5p | 5256 | 5011 | 4934 | 4463 | 0.95 | 0.94 | 0.85 | 1.11 |  |  |  |  |
| 52. | ath-miR397a | 24 | 27 | 27 | 27 | 1.09 | 1.10 | 1.10 | 1.00 |  |  |  |  |
| 53. | ath-miR398a-5p | 63 | 52 | 62 | 66 | 0.81 | 0.98 | 1.04 | 0.93 |  |  |  |  |
| 54. | ath-miR398b-3p | 12589 | 11727 | 12990 | 12248 | 0.93 | 1.03 | 0.97 | 1.06 |  |  |  |  |
| 55. | ath-miR398b-5p | 186 | 178 | 244 | 192 | 0.96 | 1.31 | 1.03 | 1.27 |  |  |  |  |
| 56. | ath-miR398c-3p | 12589 | 11727 | 12990 | 12248 | 0.93 | 1.03 | 0.97 | 1.06 |  |  |  |  |
| 57. | ath-miR398c-5p | 186 | 178 | 244 | 192 | 0.96 | 1.31 | 1.03 | 1.27 |  |  |  |  |
| 59. | ath-miR399a | 298 | 258 | 287 | 417 | 0.87 | 0.96 | 1.40 | 0.69 |  |  |  | * |
| 58. | ath-miR399b | 44 | 30 | 52 | 121 | 0.69 | 1.20 | 2.78 | 0.43 |  |  | * | * |
| 60. | ath-miR399c-3p | 304 | 261 | 289 | 420 | 0.86 | 0.95 | 1.38 | 0.69 |  |  |  | * |
| 61. | ath-miR399c-5p | 5 | 5 | 4 | 10 | 0.86 | 0.66 | 1.86 | 0.40 |  |  |  | * |
| 62. | ath-miR402 | 3 | 2 | 2 | 3 | 0.95 | 0.80 | 1.07 | 0.74 |  |  |  |  |
| 63. | ath-miR403-3p | 4240 | 4462 | 5047 | 4491 | 1.05 | 1.19 | 1.06 | 1.12 |  |  |  |  |
| 64. | ath-miR403-5p | 17 | 10 | 11 | 12 | 0.60 | 0.64 | 0.74 | 0.87 | * |  |  |  |
| 65. | ath-miR472-3p | 87 | 90 | 76 | 69 | 1.03 | 0.87 | 0.79 | 1.10 |  |  |  |  |
| 66. | ath-miR472-5p | 9 | 7 | 12 | 10 | 0.81 | 1.35 | 1.12 | 1.20 |  |  |  |  |
| 67. | ath-miR5012 | 4 | 3 | 5 | 6 | 0.87 | 1.43 | 1.58 | 0.91 |  |  |  |  |
| 68. | ath-miR5642a | 125 | 99 | 80 | 91 | 0.79 | 0.64 | 0.73 | 0.87 |  | * |  |  |
| 69. | ath-miR5642b | 125 | 99 | 80 | 91 | 0.79 | 0.64 | 0.73 | 0.87 |  | * |  |  |
| 70. | ath-miR5643a | 43 | 35 | 29 | 32 | 0.81 | 0.66 | 0.74 | 0.89 |  | * |  |  |
| 71. | ath-miR5643b | 46 | 39 | 31 | 34 | 0.85 | 0.67 | 0.75 | 0.89 |  | * |  |  |
| 72. | ath-miR5644 | 29 | 20 | 16 | 17 | 0.69 | 0.56 | 0.59 | 0.95 |  | ** | * |  |
| 73. | ath-miR5645a | 3 | 2 | 3 | 1 | 0.69 | 0.90 | 0.34 | 2.65 |  |  | * |  |
| 74. | ath-miR5645d | 3 | 2 | 3 | 1 | 0.69 | 0.90 | 0.34 | 2.65 |  |  | * |  |
| 75. | ath-miR5645e | 3 | 2 | 3 | 1 | 0.69 | 0.90 | 0.34 | 2.65 |  |  | * |  |
| 76. | ath-miR5645f | 3 | 2 | 3 | 1 | 0.69 | 0.90 | 0.34 | 2.65 |  |  | * |  |
| 77. | ath-miR5648-5p | 2 | 1 | 1 | 1 | 0.54 | 0.48 | 0.75 | 0.65 |  |  |  |  |
| 78. | ath-miR5653 | 59 | 39 | 37 | 41 | 0.67 | 0.62 | 0.71 | 0.89 |  | * |  |  |
| 79. | ath-miR5663-5p | 3 | 5 | 6 | 6 | 1.44 | 1.95 | 1.78 | 1.10 |  | * | * |  |
| 80. | ath-miR5995b | 1 | 2 | 1 | 1 | 1.45 | 0.78 | 0.97 | 0.81 |  |  |  |  |
| 81 | ath-miR773a | 52 | 42 | 40 | 47 | 0.81 | 0.76 | 0.91 | 0.84 |  |  |  |  |
| 82 | ath-miR780.1 | 8 | 11 | 6 | 6 | 1.39 | 0.69 | 0.75 | 0.92 |  |  |  |  |
| 83 | ath-miR780.2 | 432 | 407 | 317 | 324 | 0.94 | 0.73 | 0.75 | 0.98 |  |  |  |  |
| 84 | ath-miR8167a | 20 | 13 | 9 | 13 | 0.65 | 0.47 | 0.62 | 0.75 |  | * |  |  |
| 85 | ath-miR8167b | 20 | 13 | 9 | 13 | 0.65 | 0.47 | 0.62 | 0.75 |  | * |  |  |
| 86 | ath-miR8167c | 20 | 13 | 9 | 13 | 0.65 | 0.47 | 0.62 | 0.75 |  | * |  |  |
| 87 | ath-miR8167d | 20 | 13 | 9 | 13 | 0.65 | 0.47 | 0.62 | 0.75 |  | * |  |  |
| 88 | ath-miR8167e | 20 | 13 | 9 | 13 | 0.65 | 0.47 | 0.62 | 0.75 |  | * |  |  |
| 89 | ath-miR8167f | 20 | 13 | 9 | 13 | 0.65 | 0.47 | 0.62 | 0.75 |  | * |  |  |
| 90 | ath-miR8172 | 9 | 6 | 4 | 6 | 0.68 | 0.45 | 0.71 | 0.64 |  | * |  |  |
| 91 | ath-miR8175 | 107 | 142 | 154 | 93 | 1.32 | 1.44 | 0.87 | 1.66 |  | * |  |  |
| 92 | ath-miR822-3p | 9 | 9 | 9 | 8 | 0.91 | 0.93 | 0.86 | 1.08 |  |  |  |  |
| 93 | ath-miR822-5p | 83 | 87 | 93 | 79 | 1.04 | 1.12 | 0.95 | 1.18 |  |  |  |  |
| 94 | ath-miR824-3p | 304 | 285 | 340 | 328 | 0.94 | 1.12 | 1.08 | 1.04 |  |  |  |  |
| 95 | ath-miR825 | 33 | 29 | 23 | 20 | 0.89 | 0.71 | 0.62 | 1.15 |  |  | * |  |
| 96 | ath-miR827 | 274 | 234 | 318 | 538 | 0.86 | 1.16 | 1.97 | 0.59 |  |  |  |  |
| 97 | ath-miR829-3p.1 | 8 | 7 | 4 | 5 | 0.84 | 0.51 | 0.57 | 0.90 |  | * |  |  |
| 98 | ath-miR839-5p | 10 | 7 | 9 | 11 | 0.70 | 0.90 | 1.03 | 0.87 |  |  |  |  |
| 99 | ath-miR840-3p | 12 | 16 | 12 | 16 | 1.30 | 1.00 | 1.31 | 0.76 |  |  |  | * |
| 100 | ath-miR842 | 1 | 2 | 3 | 1 | 1.80 | 2.39 | 0.53 | 4.52 |  |  |  | * |
| 101 | ath-miR843 | 4 | 10 | 6 | 6 | 2.43 | 1.62 | 1.51 | 1.07 | * |  |  |  |
| 102 | ath-miR846-5p | 10 | 5 | 8 | 8 | 0.54 | 0.85 | 0.85 | 1.00 | * |  |  |  |
| 103 | ath-miR858a | 17 | 18 | 21 | 22 | 1.04 | 1.18 | 1.25 | 0.94 |  |  |  |  |
| 104 | ath-miR860 | 7 | 4 | 5 | 4 | 0.51 | 0.67 | 0.52 | 1.28 |  |  | * |  |
| 105 | ath-miR863-3p | 13 | 14 | 15 | 14 | 1.04 | 1.13 | 1.07 | 1.06 |  |  |  |  |
| 106 | ath-miR869.2 | 6 | 7 | 8 | 6 | 1.11 | 1.28 | 0.94 | 1.36 |  |  |  |  |
|  |  |  |  |  |  |  |  |  |  |  |  |  |  |

The abundance of all miRNAs normalized to transcript expression levels per million counts (CPM). miRNA expression level was calculated according to the formula, fold change = (normalized CPM)_treatment 1_/(normalized CPM)_treatment 2_. Highly significant fold change values were represented by ** ( *p* value ≤ 0.01) and those of lesser significance by * (0.05≥ *p* value< 0.01).
