# Supplementary material for: Ascophyllum nodosum extract mitigates salinity stress in Arabidopsis thaliana by modulating the expression of miRNA involved in stress tolerance and nutrient acquisition
Source: PLoS One. 2018 Oct 29;13(10):e0206221. doi: 10.1371/journal.pone.0206221 (PMC6205635; doi:10.1371/journal.pone.0206221)
Supplement: S4 Table — (DOCX) [file pone.0206221.s013.docx]

|  | **S4 Table.**  **The differential expression of conserved *Arabidopsis* miRNAs among control (C), ANE (T_1_) and ANE+NaCl (T_2_) and NaCl (T_3_) treatments at 12 h.** | | | | | | | | | | | | |
| --- | --- | --- | --- | --- | --- | --- | --- | --- | --- | --- | --- | --- | --- |
| S.No. | miRNA family | Average number of reads per library | | | | Fold Change | | | | Significant change | | | |
|  |  | Control (C) | ANE  (T_1_) | ANE+  NaCl (T_2_) | NaCl (T_3_) | T_1_ Vs C | T_2_ Vs C | T_3_ Vs C | T_2_ Vs T_3_ | T_1_ Vs C | T_2_ Vs C | T_3_ Vs C | T_2_ Vs T_3_ |
| 1. | ath-miR156g | 2.31 | 2.21 | 3.54 | 1.36 | 0.954 | 1.532 | 0.587 | 2.610 |  | * |  | * |
| 2. | ath-miR156h | 8.88 | 13.58 | 11.38 | 10.49 | 1.529 | 1.281 | 1.182 | 1.084 | * |  |  |  |
| 3. | ath-miR156j | 14.26 | 12.54 | 12.84 | 10.99 | 0.880 | 0.901 | 0.771 | 1.168 |  |  |  |  |
| 4. | ath-miR157a-3p | 34.24 | 33.31 | 31.77 | 23.61 | 0.973 | 0.928 | 0.690 | 1.346 |  |  |  |  |
| 5. | ath-miR157a-5p | 3358.03 | 2781.96 | 2398.96 | 2117.26 | 0.828 | 0.714 | 0.631 | 1.133 |  |  | ** |  |
| 6. | ath-miR157b-3p | 34.24 | 33.31 | 31.77 | 23.61 | 0.973 | 0.928 | 0.690 | 1.346 |  |  |  |  |
| 7. | ath-miR157b-5p | 3358.03 | 2781.96 | 2398.96 | 2117.26 | 0.828 | 0.714 | 0.631 | 1.133 |  |  | ** |  |
| 8. | ath-miR157c-3p | 304.28 | 248.63 | 219.67 | 187.70 | 0.817 | 0.722 | 0.617 | 1.170 |  |  | * |  |
| 9. | ath-miR157c-5p | 4330.46 | 3561.99 | 3130.15 | 2738.51 | 0.823 | 0.723 | 0.632 | 1.143 |  |  | ** |  |
| 10. | ath-miR157d | 256.22 | 222.59 | 254.51 | 234.61 | 0.869 | 0.993 | 0.916 | 1.085 |  |  |  |  |
| 11. | ath-miR158a-5p | 74.40 | 54.13 | 45.75 | 43.12 | 0.728 | 0.615 | 0.580 | 1.061 |  |  | * |  |
| 12. | ath-miR158b | 137.27 | 170.18 | 174.35 | 138.82 | 1.240 | 1.270 | 1.011 | 1.256 | * |  | * |  |
| 13. | ath-miR159a | 11531.48 | 11068.80 | 13158.22 | 12018.19 | 0.960 | 1.141 | 1.042 | 1.095 |  | ** | ** |  |
| 14. | ath-miR159b-5p | 0.94 | 2.60 | 1.08 | 0.96 | 2.757 | 1.146 | 1.017 | 1.127 | * |  |  |  |
| 15. | ath-miR160c-3p | 33.11 | 23.53 | 32.48 | 33.35 | 0.711 | 0.981 | 1.007 | 0.974 |  |  |  |  |
| 16. | ath-miR164c-3p | 2.41 | 2.17 | 4.55 | 2.61 | 0.900 | 1.890 | 1.084 | 1.743 |  |  |  |  |
| 17. | ath-miR165a-3p | 37930.26 | 35966.32 | 33394.83 | 35635.97 | 0.948 | 0.880 | 0.940 | 0.937 |  |  |  | * |
| 18. | ath-miR165b | 36915.96 | 35100.11 | 32460.65 | 34705.73 | 0.951 | 0.879 | 0.940 | 0.935 |  |  |  | * |
| 19. | ath-miR166a-3p | 114090.94 | 115550.05 | 114471.25 | 117297.93 | 1.013 | 1.003 | 1.028 | 0.976 |  |  | ** |  |
| 20. | ath-miR166b-3p | 106343.47 | 108296.35 | 107627.04 | 110503.37 | 1.018 | 1.012 | 1.039 | 0.974 |  |  | ** |  |
| 21. | ath-miR166c | 106412.61 | 108347.02 | 107683.71 | 110561.24 | 1.018 | 1.012 | 1.039 | 0.974 |  |  | ** |  |
| 22. | ath-miR166d | 106406.82 | 108344.30 | 107679.04 | 110557.73 | 1.018 | 1.012 | 1.039 | 0.974 |  |  | ** |  |
| 23. | ath-miR166e-3p | 106296.60 | 108253.82 | 107593.94 | 110455.15 | 1.018 | 1.012 | 1.039 | 0.974 |  |  | ** |  |
| 24. | ath-miR166f | 106297.35 | 108253.82 | 107594.51 | 110456.34 | 1.018 | 1.012 | 1.039 | 0.974 |  |  | ** |  |
| 25. | ath-miR166g | 106360.42 | 108303.41 | 107647.20 | 110510.19 | 1.018 | 1.012 | 1.039 | 0.974 |  |  | ** |  |
| 26. | ath-miR167a-3p | 2703.84 | 2645.18 | 2541.62 | 1881.09 | 0.978 | 0.940 | 0.696 | 1.351 |  |  |  |  |
| 27. | ath-miR167d | 950.29 | 857.66 | 717.81 | 645.81 | 0.903 | 0.755 | 0.680 | 1.111 |  |  |  |  |
| 28. | ath-miR168a-3p | 624.82 | 777.12 | 867.21 | 589.41 | 1.244 | 1.388 | 0.943 | 1.471 | * | ** |  | * |
| 29. | ath-miR168b-3p | 40.16 | 47.69 | 51.07 | 46.69 | 1.188 | 1.272 | 1.163 | 1.094 | * |  | * |  |
| 30. | ath-miR169a-3p | 47.06 | 29.78 | 28.46 | 21.17 | 0.633 | 0.605 | 0.450 | 1.344 | * |  | * |  |
| 31. | ath-miR169f-3p | 42.32 | 38.47 | 36.14 | 19.10 | 0.909 | 0.854 | 0.451 | 1.892 |  |  |  | * |
| 32. | ath-miR169g-5p | 1.44 | 2.67 | 3.46 | 2.39 | 1.856 | 2.403 | 1.659 | 1.449 |  | * |  |  |
| 33. | ath-miR169h | 67.25 | 55.12 | 52.37 | 40.61 | 0.820 | 0.779 | 0.604 | 1.290 |  |  | * |  |
| 34. | ath-miR169i | 67.72 | 56.54 | 53.83 | 40.82 | 0.835 | 0.795 | 0.603 | 1.319 |  |  | * |  |
| 35. | ath-miR169k | 67.25 | 55.12 | 52.37 | 40.61 | 0.820 | 0.779 | 0.604 | 1.290 |  |  | * |  |
| 36. | ath-miR169m | 69.73 | 57.35 | 53.03 | 41.39 | 0.823 | 0.761 | 0.594 | 1.281 |  |  | * |  |
| 37. | ath-miR171a-5p | 53.45 | 46.51 | 34.10 | 33.89 | 0.870 | 0.638 | 0.634 | 1.006 |  |  |  |  |
| 38. | ath-miR171b-5p | 29.16 | 26.17 | 25.59 | 21.28 | 0.897 | 0.877 | 0.730 | 1.203 |  |  |  |  |
| 39. | ath-miR171c-5p | 24.62 | 19.46 | 13.63 | 12.69 | 0.790 | 0.554 | 0.515 | 1.074 |  |  | * |  |
| 40. | ath-miR173-5p | 38.62 | 24.39 | 21.16 | 19.58 | 0.631 | 0.548 | 0.507 | 1.080 |  |  | * |  |
| 41. | ath-miR1888a | 1.84 | 3.35 | 2.69 | 3.45 | 1.816 | 1.458 | 1.871 | 0.779 |  |  | * |  |
| 42. | ath-miR2111b-3p | 6.66 | 6.37 | 5.99 | 6.03 | 0.956 | 0.898 | 0.905 | 0.992 |  |  |  |  |
| 43. | ath-miR2933a | 2.84 | 1.67 | 2.43 | 3.14 | 0.589 | 0.857 | 1.105 | 0.776 |  |  |  |  |
| 44. | ath-miR391-3p | 290.13 | 222.37 | 184.84 | 149.57 | 0.766 | 0.637 | 0.516 | 1.236 |  | * | ** |  |
| 45. | ath-miR393a-5p | 367.43 | 250.36 | 229.41 | 207.13 | 0.681 | 0.624 | 0.564 | 1.108 | * | * | ** |  |
| 46. | ath-miR393b-5p | 367.43 | 250.36 | 229.41 | 207.13 | 0.681 | 0.624 | 0.564 | 1.108 | * | * | ** |  |
| 47. | ath-miR395b | 4.38 | 5.89 | 6.41 | 5.10 | 1.344 | 1.463 | 1.164 | 1.256 |  |  |  |  |
| 48. | ath-miR395c | 4.38 | 5.89 | 6.41 | 5.10 | 1.344 | 1.463 | 1.164 | 1.256 |  |  |  |  |
| 49. | ath-miR395f | 4.38 | 5.89 | 6.41 | 5.10 | 1.344 | 1.463 | 1.164 | 1.256 |  |  |  |  |
| 50. | ath-miR396a-5p | 3344.73 | 3213.45 | 3089.75 | 2348.40 | 0.961 | 0.924 | 0.702 | 1.316 |  |  | * | * |
| 51. | ath-miR396b-5p | 4403.81 | 4556.53 | 3600.67 | 3383.41 | 1.035 | 0.818 | 0.768 | 1.064 |  |  |  |  |
| 52. | ath-miR397a | 35.65 | 31.22 | 27.92 | 17.67 | 0.876 | 0.783 | 0.496 | 1.580 |  |  | ** |  |
| 53. | ath-miR398a-5p | 59.54 | 67.47 | 48.83 | 54.04 | 1.133 | 0.820 | 0.908 | 0.904 |  |  |  |  |
| 54. | ath-miR398b-3p | 11552.59 | 10681.44 | 13078.28 | 11252.54 | 0.925 | 1.132 | 0.974 | 1.162 |  |  | * |  |
| 55. | ath-miR398b-5p | 205.40 | 183.46 | 293.23 | 236.42 | 0.893 | 1.428 | 1.151 | 1.240 |  | ** | ** |  |
| 56. | ath-miR398c-3p | 11552.59 | 10681.44 | 13078.28 | 11252.54 | 0.925 | 1.132 | 0.974 | 1.162 |  |  | * |  |
| 57. | ath-miR398c-5p | 205.40 | 183.46 | 293.23 | 236.42 | 0.893 | 1.428 | 1.151 | 1.240 |  | ** |  |  |
| 59. | ath-miR399a | 225.42 | 203.74 | 223.03 | 246.07 | 0.904 | 0.989 | 1.092 | 0.906 |  |  |  |  |
| 58. | ath-miR399b | 19.45 | 29.14 | 18.97 | 51.69 | 1.498 | 0.975 | 2.657 | 0.367 |  |  | ** | ** |
| 60. | ath-miR399c-3p | 226.58 | 207.91 | 224.65 | 246.29 | 0.918 | 0.991 | 1.087 | 0.912 |  |  |  | * |
| 61. | ath-miR399c-5p | 3.00 | 2.78 | 1.50 | 1.89 | 0.926 | 0.499 | 0.628 | 0.794 |  |  |  |  |
| 62. | ath-miR402 | 0 | 0 | 2.08 | 1.78 | 0 | 0 | 0 | 1.169 |  |  |  |  |
| 63. | ath-miR403-3p | 5611.66 | 4895.59 | 4596.38 | 3697.15 | 0.872 | 0.819 | 0.659 | 1.243 |  |  | * |  |
| 64. | ath-miR403-5p | 15.70 | 9.33 | 9.91 | 10.12 | 0.594 | 0.631 | 0.645 | 0.979 |  |  |  |  |
| 65. | ath-miR472-3p | 85.53 | 66.44 | 63.84 | 53.16 | 0.777 | 0.746 | 0.621 | 1.201 |  |  | * |  |
| 66. | ath-miR472-5p | 9.20 | 10.68 | 8.63 | 8.03 | 1.161 | 0.938 | 0.872 | 1.075 |  |  |  |  |
| 67. | ath-miR5012 | 7.20 | 4.80 | 2.79 | 3.30 | 0.667 | 0.388 | 0.459 | 0.845 |  | * | * |  |
| 68. | ath-miR5642a | 99.21 | 83.05 | 82.22 | 74.22 | 0.837 | 0.829 | 0.748 | 1.108 |  |  |  |  |
| 69. | ath-miR5642b | 99.21 | 83.05 | 82.22 | 74.22 | 0.837 | 0.829 | 0.748 | 1.108 |  |  |  |  |
| 70. | ath-miR5643a | 30.13 | 29.24 | 28.80 | 29.15 | 0.971 | 0.956 | 0.968 | 0.988 |  |  |  |  |
| 71. | ath-miR5643b | 32.63 | 30.54 | 30.36 | 30.26 | 0.936 | 0.930 | 0.927 | 1.003 |  |  |  |  |
| 72. | ath-miR5644 | 28.10 | 19.37 | 16.45 | 19.19 | 0.689 | 0.585 | 0.683 | 0.857 |  | * |  |  |
| 73. | ath-miR5645a | 3.50 | 2.16 | 2.56 | 3.34 | 0.617 | 0.731 | 0.956 | 0.765 |  |  |  |  |
| 74. | ath-miR5645d | 3.50 | 2.16 | 2.56 | 3.34 | 0.617 | 0.731 | 0.956 | 0.765 |  |  |  |  |
| 75. | ath-miR5645e | 3.50 | 1.92 | 2.56 | 2.95 | 0.548 | 0.731 | 0.843 | 0.867 |  |  |  |  |
| 76. | ath-miR5645f | 3.50 | 2.16 | 2.56 | 3.34 | 0.617 | 0.731 | 0.956 | 0.765 |  |  |  |  |
| 77. | ath-miR5648-5p | 3.31 | 0.95 | 0.74 | 1.26 | 0.287 | 0.222 | 0.380 | 0.585 | * | * |  |  |
| 78. | ath-miR5653 | 50.88 | 34.92 | 37.46 | 38.72 | 0.686 | 0.736 | 0.761 | 0.967 |  |  |  |  |
| 79. | ath-miR5663-5p | 6.78 | 3.62 | 4.79 | 4.43 | 0.533 | 0.705 | 0.653 | 1.081 |  |  |  |  |
| 80. | ath-miR5995b | 3.00 | 1.33 | 0.91 | 0.87 | 0.442 | 0.302 | 0.290 | 1.043 |  |  | * |  |
| 81. | ath-miR773a | 50.45 | 31.83 | 32.54 | 30.36 | 0.631 | 0.645 | 0.602 | 1.072 | * |  |  |  |
| 82. | ath-miR780.1 | 8.95 | 11.32 | 12.15 | 13.32 | 1.265 | 1.358 | 1.488 | 0.912 |  |  | * |  |
| 83. | ath-miR780.2 | 354.60 | 347.17 | 407.47 | 435.17 | 0.979 | 1.149 | 1.227 | 0.936 |  |  | ** |  |
| 84. | ath-miR8167a | 20.73 | 10.48 | 5.29 | 15.47 | 0.506 | 0.255 | 0.746 | 0.342 | * | ** |  | ** |
| 85. | ath-miR8167b | 20.73 | 10.48 | 5.29 | 15.47 | 0.506 | 0.255 | 0.746 | 0.342 | * | ** |  | ** |
| 86. | ath-miR8167c | 20.73 | 10.48 | 5.29 | 15.47 | 0.506 | 0.255 | 0.746 | 0.342 | * | ** |  | ** |
| 87. | ath-miR8167d | 20.73 | 10.48 | 5.29 | 15.47 | 0.506 | 0.255 | 0.746 | 0.342 | * | ** |  | ** |
| 88. | ath-miR8167e | 20.73 | 10.48 | 5.29 | 15.47 | 0.506 | 0.255 | 0.746 | 0.342 | * | ** |  | ** |
| 89. | ath-miR8167f | 20.73 | 10.48 | 5.29 | 15.47 | 0.506 | 0.255 | 0.746 | 0.342 | * | ** |  | ** |
| 90. | ath-miR8172 | 4.88 | 5.02 | 5.16 | 5.40 | 1.029 | 1.058 | 1.106 | 0.956 |  |  |  |  |
| 91. | ath-miR8175 | 55.79 | 63.22 | 137.40 | 118.35 | 1.133 | 2.463 | 2.121 | 1.161 |  | ** | ** |  |
| 92. | ath-miR822-3p | 12.46 | 7.61 | 5.22 | 6.79 | 0.611 | 0.419 | 0.545 | 0.768 |  | ** |  |  |
| 93. | ath-miR822-5p | 102.46 | 87.20 | 70.42 | 60.67 | 0.851 | 0.687 | 0.592 | 1.161 |  |  | * |  |
| 94. | ath-miR824-3p | 330.83 | 292.82 | 379.66 | 343.47 | 0.885 | 1.148 | 1.038 | 1.105 |  |  | * |  |
| 95. | ath-miR825 | 22.97 | 19.18 | 16.12 | 17.56 | 0.835 | 0.702 | 0.765 | 0.918 |  |  |  |  |
| 96. | ath-miR827 | 145.04 | 189.32 | 184.40 | 324.78 | 1.305 | 1.271 | 2.239 | 0.568 |  |  | ** | ** |
| 97. | ath-miR829-3p.1 | 8.01 | 7.67 | 5.06 | 6.15 | 0.957 | 0.632 | 0.768 | 0.823 |  |  |  |  |
| 98. | ath-miR839-5p | 10.30 | 8.52 | 7.82 | 12.38 | 0.827 | 0.759 | 1.202 | 0.631 |  |  |  | * |
| 99. | ath-miR840-3p | 18.56 | 11.46 | 11.67 | 11.37 | 0.618 | 0.629 | 0.613 | 1.026 | * |  |  |  |
| 100 | ath-miR842 | 2.79 | 1.03 | 2.34 | 1.83 | 0.371 | 0.841 | 0.656 | 1.282 |  |  |  |  |
| 101 | ath-miR843 | 6.36 | 2.66 | 6.40 | 7.09 | 0.418 | 1.007 | 1.116 | 0.902 | * |  |  |  |
| 102 | ath-miR846-5p | 11.70 | 8.30 | 11.04 | 8.85 | 0.710 | 0.944 | 0.757 | 1.247 |  |  |  |  |
| 103 | ath-miR858a | 16.69 | 15.77 | 22.70 | 20.02 | 0.945 | 1.360 | 1.199 | 1.134 |  |  |  |  |
| 104 | ath-miR860 | 4.88 | 5.44 | 3.11 | 3.99 | 1.114 | 0.637 | 0.817 | 0.780 |  |  |  |  |
| 105 | ath-miR863-3p | 16.27 | 10.32 | 12.19 | 7.74 | 0.634 | 0.749 | 0.476 | 1.575 |  |  | * |  |
| 106 | ath-miR869.2 | 6.16 | 6.96 | 6.53 | 3.94 | 1.129 | 1.060 | 0.639 | 1.658 |  |  |  |  |

The abundance of all miRNAs normalized to transcript expression levels per million counts (CPM). miRNA expression level was calculated according to the formula, fold change = (normalized CPM)_treatment 1_/(normalized CPM)_treatment 2_. Highly significant fold change values were represented by ** ( *p* value ≤ 0.01) and those of lesser significance by * (0.05≥ *p* value< 0.01).
