# Supplementary material for: Ascophyllum nodosum extract mitigates salinity stress in Arabidopsis thaliana by modulating the expression of miRNA involved in stress tolerance and nutrient acquisition
Source: PLoS One. 2018 Oct 29;13(10):e0206221. doi: 10.1371/journal.pone.0206221 (PMC6205635; doi:10.1371/journal.pone.0206221)
Supplement: S5 Table — (DOCX) [file pone.0206221.s014.docx]

**S5 Table. List of target genes predicted.**

| **Family** | **Target genes** | **Potential role of target gene** | **Inhibition** | **Expectation score** |
| --- | --- | --- | --- | --- |
| ath-miR842 | Mannose-binding lectin superfamily protein (At1g52120, At5g38550, At1g57570, At1g60130) | Carbohydrate binding, interacting selectively and non-covalently with any carbohydrate | Cleavage | 1.5-2.0 |
| ath-miR5645a,d,e,f | Pyridoxal phosphate (PLP)-dependent transferases superfamily protein (At4g33680), Leucine Rich Repeat domains containing protein (At5g22600) | At4g33680: involved in disease resistance against *Pseudomonas syringae* | Cleavage | 3.0 |
| ath-miR395b,c,f | sulphate transporter 2;1 (At5g10180), ATP sulfurylase1 (At3g22890), ATP sulfurylase3 (At4g14680), ATP sulfurylase4 (At5g43780), ABA binding protein (ABAR, CCH, CCH1, CHLH) (At5g13630) | At5g10180, At3g22890, At4g14680, At5g43780: Regulation of sulphate accumulation, Magnesium chelatase involved in plastid to nucleus signal transduction | Cleavage | 3.0 |
| ath-miR8175 | Galactose oxidase/kelch repeat superfamily protein (At1g22040) | involved in: n-terminal protein myristoylation | Translation | 3.0 |
| ath-miR168a | Argonaute 1 (At1g48410.3), Set domain protein 31; SDG31 (At3g04380.1) | At1g48410.3: protein regulates genes involved at the cross talk between auxin and light signaling during adventitious root development; At3g04380.1: histone lysine methylation | Cleavage | 3.0 |
| ath-miR860 | RNA binding (RRM/RBD/RNP motifs) family protein (At3g12640.1) | At3g12640.1: involved in the conversion of a primary mRNA transcript into one or more mature mRNA(s) prior to translation into polypeptide (mRNA maturation) | Cleavage | 2.5 |
| ath-miR398b,c | Superoxide dismutase 1 (ATSOD1, CSD1, SOD1) (At1g08830.1)(Induction of SOD is downregulated by miRNA) | At1g08830.1: Encodes a cytosolic copper/zinc superoxide dismutase CSD1 that can detoxify superoxide radicals | Cleavage | 3.0 |
| ath-miR169a, h,i,k,f,m | nuclear factor Y, subunit A8" (NF-YA8**)** (At1g17590), nuclear factor Y, subunit A5 (At1g54160), nuclear factor Y, subunit A3 (ATHAP2C, HAP2C, NF-YA3) (At1g72830), nuclear factor Y, subunit A2, (ATHAP2B, HAP2B, NF-YA2, UNE8) (ATHAP2B, HAP2B, NF-YA2, UNE8), CCAAT Binding Factor (CBF) and HAP2-like transcription factors | At1g17590, At1g54160, At1g72830: functions in sequence-specific DNA binding transcription factor activity | Cleavage | 3.0 |
| ath-miR396a | **Direct target:** growth-regulating factors, AtGRF1, AtGRF3, AtGRF5, AtGRF6, AtGRF9, AtGRF4, AtGRF8, AtGRF2, AtGRF7 (At2g06200, At2g22840, At2g36400, At2g45480, At3g13960, At3g52910, At4g24150, At4g37740, At5g53660), Pentatricopeptide repeat (PPR) superfamily protein (At2g15630), ALC-interacting protein 1 (At5g01370)  **Indirect target:** AtGRF7 regulates transcriptional repressor of ABA and osmotic stress responsive genes including DREB2a (At5g05410), heat shock transcription factor A3 (At5g03720), desiccation responsive protein 29A (RD29A) (At5g52310) | At2g06200, At2g22840, At2g36400, At2g45480, At3g13960, At3g52910, At4g24150, At4g37740, At5g53660: Growth regulating factor encoding transcription activator | Cleavage | 2.0 |
| ath-miR158b | Dof-type zinc finger DNA-binding family protein (At2g46590.2) | At2g46590.2: Encode for protein Dof zinc finger motifs involved in seed germination, response to light and cold treatment | Translation | 3.0 |
| ath-miR399b,c | wall-associated kinase 2 (At1g21270), phosphate 2 (ATUBC24, PHO2, UBC24) (At2g33770) | At1g21270: Cytoplasmic serine/threonine protein kinase induced by salicylic acid, At2g33770: encodes an ubiquitin-conjugating E2 enzyme. UBC24 mRNA accumulation is suppressed by miR399f, miR399b and miR399c. | Cleavage | 1.5, 3.0 |
| ath-miR399a | Terpenoid cyclases family protein, PEN6 (At1g78500),  phosphate 2 (ATUBC24, PHO2, UBC24) (At2g33770),  cytochrome P450, family 705, subfamily A, polypeptide 30 (At3g20940) | At1g78500: pentacyclic triterpenoid biosynthetic process,  At2g33770:inorganic phosphate (Pi) homeostasis,  At3g20940: involved in oxidation-reduction process and secondary metabolite biosynthetic process, | Cleavage | 3.0 |
| ath-miR169g | nuclear factor Y, subunit A2 (ATHAP2B, HAP2B, NF-YA2, UNE8), (At3g05690), nuclear factor Y, subunit A9 (At3g20910, NF-YA9), nuclear factor Y, subunit A10 (At5g06510, NF-YA10), nuclear factor Y, subunit A1 (At5g12840, ATHAP2A, EMB2220, HAP2A, NF-YA1),  Thioredoxin superfamily protein (At1g20225), high-affinity nickel-transport family protein (At4g35080) | At3g05690, At3g20910, Encodes a subunit of CCAAT-binding complex, binds to CCAAT box motif present in some plant promoter sequences,  Nickel cation transport, | Cleavage | 3.0 |
| ath-miR827 | SPX (SYG1/Pho81/XPR1) domain-containing protein (BAH1, NLA) (At1g02860) | Encodes a ubiquitin E3 ligase with RING and SPX domains that is involved in mediating immune responses and mediates degradation of PHT1s at plasma membranes | Cleavage | 3.0 |
| ath-miR5648 | ABCC1, *Arabidopsis* *thaliana* ATP-binding cassette C1 (At1g30400.1), ABCC2, *Arabidopsis* *thaliana* ATP-binding cassette C2, (At2g34660, ATMRP2, EST4, MRP2), embryo sac development arrest 16 (EDA16) (At1g61140.1), | At1g30400.1: Multidrug resistant protein,  At2g34660: Multidrug resistant protein  At1g61140.1: embryo sac development | Cleavage | 3.0 |
| ath-mir2111b | Galactose oxidase/kelch repeat superfamily protein (At3g27150.1), Calcineurin-like metallo-phosphoesterase superfamily protein (At1g07010), O-fucosyl transferase family protein (At1g11990.1), myb-like transcription factor family protein (At1g25550), NAC (No Apical Meristem) domain transcriptional regulator superfamily protein (At1g60380.1), C2H2-like zinc finger protein (At5g52010.1), ACC oxidase 1(ACO1, ATACO1, At2g19590.1), Magnesium chelatase I2 (CHL I2, CHLI-2, CHLI2, At5g45930.1) | At3g27150.1: Induced by phosphate starvation, At1g07010: protein serine/threonine phosphatase activity, At1g25550, At1g60380.1, At5g52010.1, At2g19590.1: regulation of transcription, sequence-specific DNA binding transcription factor activity, At2g19590.1: cellular response to fatty acid, At5g45930.1: chlorophyll biosynthetic process | Cleavage, Translation | 1.0-2.5 |
| ath-miR8167a-f | Major facilitator superfamily protein (At1g27040.1, AtNPF4.5, NPF4.5, NRT1/ PTR FAMILY 4.5), Cytochrome P450, family 705, subfamily a, polypeptide 15" (CYP705A15, At3g20080.3), | At1g27040.1: Involved in oligopeptide transport, At3g20080.3: secondary metabolite biosynthetic process | Cleavage | 3.0 |
| ath-miR156g | **Direct targets**: phytochelatin synthase 2 (At1g03980.1), Squamosa-promoter Binding Protein (SBP)-like transcription factors (At3g57920.1, At1g27360.2, At1g27360.3, At5g50670.1, At2g42200.1, At1g27360.1, At1g27360.4, At5g43270.3, At1g27370.3, At5g43270.2, At5g50570.2, At1g27370.4, At5g43270.1, At5g50570.1, At1g27370.2, At1g69170.1, At1g27370.1, At2g33810.1, At1g53160.1), Protein kinase superfamily protein (At3g28690.2), Galactosyltransferase family protein (At1g53290.1)  **Indirect targets:** Agamous-like 7 (AGL7, AP1, APETALA1, ATAP1, At1g69120.1, At3g54340.1),  floral defective 10 (FLO10) (At3g23130.1, At3g23130),Flowering locus T (At1g65480.1) | At1g03980.1: Phytochelatin biosynthetic process, At2g42200.1, At3g57920.1: involved in the vegetative to reproductive phase transition, At1g27360.1, At1g27360.2, At1g27360.3, At1g27370.1, At5g43270.3, At5g43270.2, , At5g43270.1, At1g27370.2, At1g27370.4, At2g33810, At1g53160.1: controls proper development of lateral organs in association with shoot maturation in the reproductive phase, At5g50570.1, At5g50570.2: Squamosa promoter-binding protein-like (SBP domain) transcription factor family protein, At3g28690.2: protein serine/threonine kinase activity, At1g53290.1: protein glycosylation  At1g69120.1, At3g54340.1: Floral homeotic gene encoding a MADS domain protein homologous to SRF transcription factors,  At3g23130.1, At3g23130, At1g65480.1: Flower-specific gene controlling the boundary of the stamen and carpel whorls  FT, together with LFY, promotes flowering | Cleavage | 3.0, 2.0 |
| ath-miR840-3p | Oligopeptide transporter (ATOPT1, At5g55930.1), Protein kinase superfamily protein (At1g79640.2), Argonaute family protein (At2g27880.1), ATU27 Arabidopsis thaliana U2 RNA gene (At5g61455), Galactosyltransferase family protein (At4g21060.1), Late embryogenesis abundant protein (LEA14, At1g01470.1) | At5g55930.1: Oligopeptide transport, At1g79640.2: protein serine/threonine kinase activity, defense responses, At2g27880.1, At5g61455: mRNA cis splicing, At4g21060.1: arabinogalactan protein metabolic process, At1g01470.1: induced in response to wounding and light stress | Cleavage | 0.0 |
| ath-miR157d | **Direct targets**: Squamosa-promoter Binding Protein (SBP)-like transcription factors (At3g57920.1, At1g27360.2, At1g27360.3, At5g50670.1, At2g42200.1, At1g27360.1, At1g27360.4, At5g43270.3, At1g27370.3, At5g43270.2, At5g50570.2, At1g27370.4, At5g43270.1, At5g50570.1, At1g27370.2, At1g69170.1, At1g27370.1, At2g33810.1, At1g53160.1),  DEA(D/H)-box RNA helicase family protein (ATRH25, STRS2, At5g08620) | Protein serine/threonine kinase activity.  At5g08620: involved in drought, salt and cold stress responses | Translation  Cleavage | 1.0  2.0 |
| ath-miR397a | AtLAC2 (Laccase) (At2g29130.1), *Arabidopsis* laccase-like multicopper oxidase 4 (AtLMCO4, At2g38080.1), | At2g29130.1:Lignin biosynthetic pathway, At2g38080.1: response to water deprivation | Cleavage | 0.5,1.0 |
| ath-miR863-3p | **Direct target:**  Homeodomain-like superfamily protein (At1g74840.1),  ATMYB66, MYB domain protein 66, WEREWOLF (At5g14750.1)  Ribosomal protein S27a / Ubiquitin family protein (At1g23410.1)  **Indirect target:**  Nuclear-localized R3-type MYB transcription factor (At2g46410.1),Glabra 2, a homeodomain protein (At1g79840.2),ATMYB23 (At5g40330),WRKY family transcription factor family protein, ATWRKY44 (At2g37260) | At1g74840.1: Response to ABA, involved in salt stress tolerance  At5g14750.1: Encoded a R2R3 MYB and involved in root and hypocotyl epidermal cell fate determination  At2g46410.1:involved in ubiquitin-dependent protein catabolic process  At1g79840.2: positive regulator of hair-cell differentiation and regulated by AtMYB66  Affects epidermal cell identity including trichomes, root hairs, and seed coat and regulated by AtMYB66. It also down-regulates seed oil contents.  At5g40330: encodes a MYB gene that induce ectopic trichome formation, regulated by AtMYB66.  At2g37260: expressed in the seed integument and endosperm | Cleavage | 3.0 |
| ath-miR846-5p | Disease resistance protein (CC-NBS-LRR class) family (At5g63020.1)  Leucine-rich repeat protein kinase family protein (At5g01950.1) | At5g63020.1: Defense response  At5g01950.1:involved in protein serine/threonine kinase activity | Translation  Cleavage | 1.5  2.0 |
| ath-miR391-3p | Brassinosteroid-signaling kinase 11, bsk11, Protein kinase protein with tetratricopeptide repeat domain (At1g50990) | Protein kinase activity (At1g50990) | Cleavage | 3.0 |
| ath-miR171b | Arabidopsis thaliana hairy meristem 1 (AtHAM1) belongs to LOM (Lost Meristems) genes, At2g45160 (LOM1), At3g60630 (LOM2) and At4g00150 (LOM3).  Succinyl-CoA ligase (At5g08300.1) | LOM1 and LOM2 promote cell differentiation at the periphery of shoot meristems and help to maintain their polar organization.  Involved in metabolic processes | Cleavage | 1.5 |
| ath-miR472-3p | Resistant to *P. syringae 5*, RPS5 (At1g12220)Disease resistance protein (CC-NBS-LRR class) family (At1g12290, At1g15890, At1g62630, At1g63360, At5g43740), Resistance silenced gene 1(RSG1) (At1g51480), Resistance silenced gene 2, RSG2 (At5g43730, At5g63020), LRR and NB-ARC domains-containing disease resistance protein (At1g61180, At1g61310, At1g61190, At1g61300, At4g10780, At5g05400) | At1g12220: Resistance gene,  At1g12290, At1g15890, At1g62630, At1g63360, At5g43740, At1g51480, At5g43730: mediates resistance against the bacterial pathogen *Pseudomonas* *syringae* At1g61180, At1g61310, At1g61190, At1g61300, At4g10780, At5g05400: defense response, apoptosis | Cleavage  Cleavage | 2.5  2.5 |
| ath-mir822 | Cysteine/Histidine-rich C1 domain family protein (At1g44020, At1g66450, At4g13130, At3g26250) | Oxidation-reduction processes | Translation | 2.0 |
